# Differential DNA methylation in infants with IgE- and non-IgE-mediated cow’s milk allergy and its association with acquired tolerance

**DOI:** 10.3389/fimmu.2025.1571987

**Published:** 2025-11-28

**Authors:** Rebeca López-Gómez, Marta Gil-Martínez, Ana Ladrón-Guevara, Jose Manuel Rodrigo-Muñoz, Clara Lorente-Sorolla, Daniel Rodríguez-González, Zahara García de Castro, Gema Guillén-Sánchez, Antonio Serrano-Santiago, Raquel Mirasierra-Pérez, José Antonio Cañas, Genoveva Del Río Camacho, Victoria Del Pozo

**Affiliations:** 1 Pediatrics Department, University Hospital Fundación Jiménez Díaz, Madrid, Spain; 2 Immunology Department, Health Research Institute-Fundación Jiménez Díaz, Universidad Autónoma de Madrid (IIS-FJD, UAM), Madrid, Spain; 3 Centro de Investigación Biomédica en Red (CIBER) de Enfermedades Respiratorias (CIBERES), Madrid, Spain; 4 Medicine Department, School of Medicine, Faculty of Medicine, Universidad Autónoma de Madrid (UAM), Madrid, Spain

**Keywords:** food allergy, cow’s milk allergy, epigenetic, methylation, IgE-mediated, exclusion diet, tolerance

## Abstract

**Background:**

Cow’s milk allergy (CMA) is one of the most common food allergies (FA) in childhood. This condition can be IgE-mediated, non-IgE-mediated, or a combination of both. Diagnosis involves clinical history in conjunction with sensitization tests. However, these tests have limited predictive value, making the oral food challenge (OFC) the gold standard for diagnosis. Recent research has focused on identifying biomarkers, including DNA methylation patterns, for FA diagnosis. The aim of this study is to investigate the differences in DNA methylation associated with distinct patterns of CMA, to identify new diagnostic biomarkers.

**Methods:**

Genome-wide DNA methylation profiling was performed on blood samples from infants with IgE-mediated CMA (CMAIE), non-IgE-mediated CMA (CMANIE), and non-allergic controls, at baseline and after 6 months of an exclusion diet in CMA groups. These results were then correlated with tolerance acquisition following the restrictive diet.

**Results:**

A total of 19 infants were enrolled (10 CMAIE, 6 CMANIE, and 3 controls). Significant differentially methylated regions (DMRs) annotated to both genes and promoters were identified in all groups, and a clear separation of the samples into their respective groups was observed. Furthermore, DMRs in promoters and genes were identified in tolerant CMAIE children after the exclusion diet, being associated to tolerance.

**Discussion:**

Differential DNA methylation in CMA children is a useful diagnostic biomarker, and it could also be valuable in predicting the resolution of such pathologies.

## 1 Introduction

Allergic diseases, including food allergies (FAs), have become increasingly prevalent in recent years. The pathogenesis of allergic disorders is complex, involving an interplay between genetic predisposition and environmental factors. Among the most common FAs, particularly in early childhood, is cow’s milk allergy (CMA), which typically manifests within the first few months of life, often following the introduction of milk-based formulas (1–3). Epidemiological data suggest that the prevalence, persistence and severity of CMA have risen significantly, resulting in a substantial negative impact on quality of life and increased medical care costs (4).

Based on the underlying immune mechanisms, CMA is classified into three types: immunoglobulin E (IgE)-mediated, non-IgE-mediated (driven by cellular immune reactions), and mixed (5). For IgE-mediated CMA, although diagnosis typically involves a combination of clinical history and sensitization tests, the oral food challenge (OFC) is the gold standard for confirming the diagnosis and is essential to determine whether the allergy has been resolved (6). In non-IgE-mediated allergy, the diagnostic criteria rely on the presence of digestive symptoms and their resolution following the elimination of the allergen from the diet (7).

In the search for alternative diagnostic methods, epigenetics has emerged as a molecular variable of interest in ongoing efforts to understand the mechanisms and outcomes of FAs, contributing factors may include lifestyle, the host microbiome, the exposome, allergy history, and delivery method (8). The interaction of these factors may induce epigenetic changes in the immune system, potentially contributing to the development of CMA (9, 10). These findings suggest that epigenetic mechanisms could serve as therapeutic targets.

Epigenetic modifications, particularly DNA methylation, have been implicated in the pathogenesis of FAs (11). DNA methylation is a well-established epigenetic mechanism that regulates gene expression by adding methyl groups, typically to a cytosine in a CpG dinucleotide, particularly in promoter regions. Classically, this restricts access for transcription, primarily in the promoter region, leading to reduced gene expression (12, 13). In the context of FAs, previous epigenome-wide association studies (EWAS) have identified DNA methylation in regions of genes implicated in FA-associated pathways (14–17). Several studies have highlighted methylation changes in specific candidate genes associated with T regulatory cell activity, as well as differential methylation of Type 1 and Type 2 cytokine genes. *Canani* et al. identified distinct DNA methylation patterns in Th1 and Th2 cytokine genes in children with CMA who developed tolerance (10). This group also observed that DNA methylation of *FOXP3* was associated with the use of hypoallergenic formula and probiotic intake in infants with CMA (18). Therefore, although DNA methylation patterns could serve as potential biomarkers, no evidence linking DNA methylation to non-IgE-mediated allergies.

Therefore, since the methylome in CMA remains largely unexplored, the aim of our study is to investigate differences in DNA methylation associated with distinct patterns of CMA, with the goal of identifying novel diagnostic biomarkers for CMA in infants. To achieve this, we used whole genome-wide methylation analysis. The results of this study will contribute to a better understanding of the epigenetic mechanisms involved in CMA and to the search for novel diagnostic biomarkers.

## 2 Materials and methods

### 2.1 Study design, subjects and variable analysis

**Figure 1:**
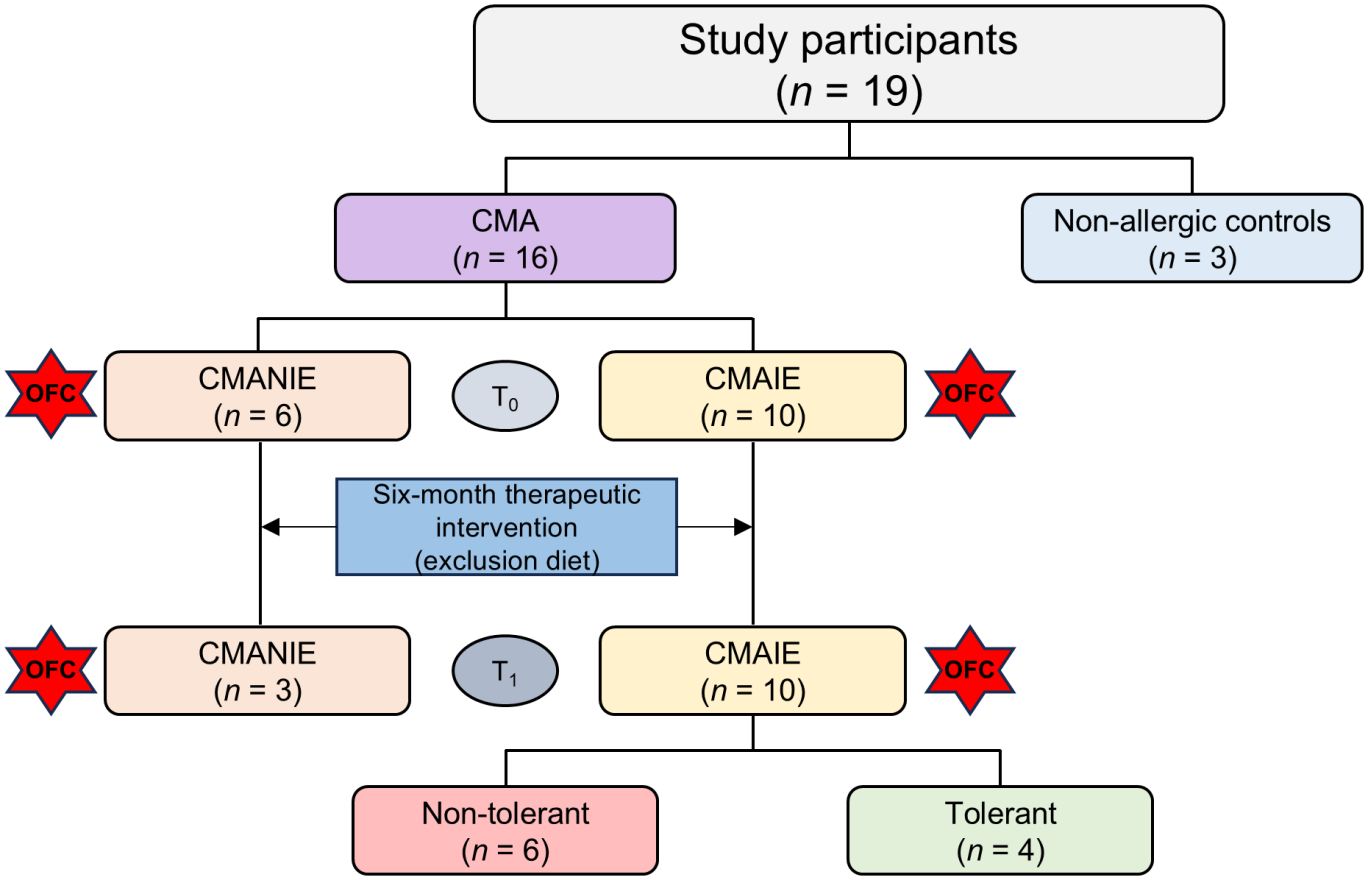
Flowchart of patient classification. Nineteen participants were recruited for the study: 3 non-allergic controls and 16 children allergic to cow’s milk protein. These patients included 6 CMANIE children and 10 CMAIE individuals at baseline (T_0_). An exclusion diet was implemented for six months, and after treatment (T_1_), the number of CMAIE participants remained the same. However, only 3 patients were recovered from the CMANIE group. In the CMAIE group, after treatment, 6 patients were non-tolerant and 4 were tolerant, as shown by the OFC results.

The study is prospective, including cases with therapeutic intervention (exclusion diet) and controls. A total of 19 participants, aged 0–12 months, referred to pediatric allergology and gastroenterology clinics, were recruited and stratified based on inclusion criteria into the following groups: patients with IgE-mediated CMA (CMAIE) (*n* = 10), patients with non-IgE-mediated CMA (CMANIE) (*n* = 6), and non-atopic infants without symptoms of CMA as the control group (*n* = 3) (**Figure 1**).

The diagnosis of CMA was made following the official national guidelines (6, 19). For IgE-mediated CMA, the diagnosis was based on symptoms consistent with an IgE-mediated allergic reaction, manifesting within two hours after ingestion of the suspected allergen, along with positive sensitization tests. The prick-by-prick test using pasteurized cow’s milk was employed, with a positive response defined as a wheal size diameter greater than 3 mm or larger than the histamine control. All participants in the CMAIE group were evaluated within one month of the allergic reaction. Given the presenting symptoms and corresponding skin test results, a controlled exposure test was not deemed necessary for confirming the diagnosis (6). In non-IgE-mediated CMA, the diagnosis was based solely on digestive symptoms (vomiting/diarrhea) occurring between 1 and 4 hours after ingestion of the food, or chronic digestive symptoms (gastroesophageal reflux, diarrhea, hematochezia, failure to thrive), with clear improvement following the removal of cow’s milk protein from the diet and negative prick-by-prick results. As an additional diagnostic criterion, for patients in the CMANIE group with mild symptoms and an uncertain diagnosis, reintroduction of the suspected food after the elimination diet could be useful. Regarding controls, children visiting our center during the same study period due to the risk of congenital infections, but without any risk of atopic disorders, were also enrolled. Infants in the CMAIE and CMANIE groups followed an exclusion diet for 6 months, after which all participants underwent a controlled milk exposure test.

Preterm infants, as well as children with eosinophilic gastrointestinal disorders, food protein-induced enterocolitis syndrome, concomitant chronic systemic diseases, immunodeficiencies, autoimmune diseases and neoplasms, were excluded from the study.

The qualitative variables collected included gender, personal history (atopic dermatitis, gastroesophageal reflux, wheezing, and other food allergies), obstetric history, family history of atopy-related conditions (atopic dermatitis, asthma, or food allergy in first-degree relatives), type of feeding (breastfeeding or formula feeding), and reported symptoms. Regarding quantitative variables, these included age at diagnosis, specific IgE (sIgE) levels to milk, and sIgE levels to milk protein fractions (casein, alpha-lactalbumin, and beta-lactoglobulin).

The study was approved by the Fundación Jiménez Díaz Ethics Committee (Madrid, Spain) and follows the principles of the Helsinki Declaration. The parents or guardians of the enrolled infants provided informed consent for participation in the study.

### 2.2 Oral food challenge

Patients, whose informed consent was signed by their parents/legal guardians, underwent a graded oral milk challenge, receiving escalating doses of milk every 30 minutes in the following sequence: 2, 5, 10, 25, 50, and 120 ml, culminating in a total dose of 212 ml. Participants were closely monitored for two hours, after completing milk intake, to evaluate the presence of immediate reactions (i.e., reactions occurring within two hours post-ingestion). The type of milk used for the test varied by age: infant formula was administered for children under 12 months, while pasteurized whole milk was used for those older than 12 months. A positive result was defined as the appearance of clinical signs indicative of an allergic reaction during or after milk exposure.

For all participants with IgE-mediated CMA and those in non-IgE-mediated CMA who exhibited moderate to severe symptoms and with a potential risk, the procedure was conducted in a hospital setting under medical supervision. In contrast, for CMANIE participants presenting with mild symptoms (e.g., proctocolitis, diarrhea, colic), the milk exposure test was conducted at home, with progressively increased doses over several weeks and with medical follow up. Resolution of allergy was determined when the exposure test yielded a negative result.

### 2.3 Sample collection and processing

A peripheral blood sample was collected from all patients at the time of recruitment (T_0_) using tubes without anticoagulants. After 6 months of the exclusion diet (T_1_), follow-up blood sampling was conducted for all participants in the CMAIE group. However, in the CMANIE group, follow-up samples were obtained from only 3 participants. For the control group, blood collection was performed only at baseline. T_1_ blood sample was obtained prior to the OFC.

Serum was separated by centrifugation at 3,000 rpm for 10 minutes at 4 °C and was subsequently used for serological analyses. These included the quantification of sIgE to whole cow’s milk proteins: α-lactoalbumin (Bos d4), β-lactoglobulin (Bos d5), and casein (Bos d8), using the ImmunoCAP assay (Phadia, Thermo Fisher Scientific, Uppsala, Sweden).

An additional fresh peripheral blood sample was collected in tubes containing EDTA as an anticoagulant. Subsequently, 1 mL of the sample was stored at -80 °C until further analysis.

### 2.4 DNA extraction and genome-wide methylation analysis

Total blood DNA was extracted using the DNA Blood Kit (Qiagen, Hilden, Germany), and DNA methylation analysis was conducted through hybridization with EPIC arrays (Illumina Inc., San Diego, CA, USA). DNA quantification was performed using the Qubit^™^ dsDNA BR Assay Kit (Invitrogen, Waltham, MA, USA). To evaluate DNA integrity as part of quality control, electrophoresis was conducted using the FlashGel^®^ System (Lonza) with 1.2% agarose DNA cassettes. The FlashGel DNA Marker (100 bp–4 kb) served as the molecular weight reference, providing defined size bands. Sodium bisulfite conversion of DNA was carried out using the EZ DNA Methylation™ Kit (Zymo Research, Irvine, CA, USA). DNA methylation analysis was subsequently performed with the Infinium Methylation EPIC-8 v2.0 Array (Illumina).

### 2.5 DNA methylation data preprocessing and quality control

Raw methylation data obtained from the arrays were processed and filtered following recommended best practices to ensure high-quality and biologically meaningful data. Specifically, the following filtering steps were applied: i) detection of p-values: probes with detection of p-values ≥ 0.01 in any sample were removed; therefore, only probes with detection p-values < 0.01 across all samples were retained; ii) sex chromosomes: probes mapping to the X and Y chromosomes were excluded to avoid potential confounding due to sex-specific methylation patterns; iii) single nucleotide polymorphisms (SNPs)-associated probes: probes with known SNPs at the CpG or single base extension (SBE) sites were excluded using the *dropLociWithSnps* function from the minfi R package (R/Bioconductor, R Foundation, Indianapolis, IN, USA), regardless of minor allele frequency; and, iv) cross-reactive probes: known cross-reactive probes, which may co-hybridize to alternate genomic sequences, were removed based on the list published by Pidsley et al. (20). After these filtering steps, the remaining probes were used for downstream analyses, ensuring that the detected methylation patterns reflect true biological variation rather than technical artifacts or underlying genetic polymorphisms.

### 2.6 Bioinformatic analysis

An initial differential methylation analysis was conducted at the single-position level using the methodology implemented in the *limma* package (R/Bioconductor), specifically designed for microarray data analysis. (21). All samples from each group were compared to identify differentially methylated CpG positions, which were considered statistically significant with a false discovery rate (FDR) less than 0.05. Following the identification of differentially methylated positions (DMPs) from the initial analysis, a subsequent differential methylation analysis at the region level (DMRs) was performed using the DMRCate package (R/Bioconductor) (22–24). DMRs with a Fisher’s adjusted p-value of less than 0.05 and a greater than 5% difference in the average methylation value between groups were considered significant.

Additionally, a DMR analysis was conducted using the Methylated CpGs Set Enrichment Analysis (mCSEA) package (R/Bioconductor) (25). This package implements a novel approach for identifying DMRs within predefined regions, such as promoters, genes, or CpG islands. Based on the Gene Set Enrichment Analysis (GSEA) method, which calculates an enrichment score (ES) using the weighted Kolmogorov-Smirnov test, which ranks differentially methylated regions (such as genes, promoters, or CpG islands). This methodology facilitates the identification of DMRs with moderate changes between study conditions. DMRs with adjusted p-value of less than 0.05 were considered statistically significant. Subsequently, a functional enrichment analysis was performed on the genes and promoters annotated to the significant DMRs identified using the mCSEA package. This analysis was conducted using the enrichR package, which interfaces with the Enrichr database (26), and included Gene Ontology (GO) terms and Kyoto Encyclopedia of Genes and Genomes (KEGG) pathways. Only enriched terms with a FDR below 0.05 were considered significant.

Moreover, for the *in silico* estimation of cellular composition in blood samples, the EpiDISH package was employed, which includes a reference matrix specifically designed for data obtained from EPIC arrays. The reference matrix used, termed cent12CT.m (R/Bioconductor), enables the estimation of relative fractions of 12 immune cell types: naïve and mature B cells, naïve and mature CD4^+^ and CD8^+^ T cells, regulatory T cells, natural killer cells, neutrophils, monocytes, eosinophils, and basophils. This function estimates cell-type proportion based on the methylation signatures of cell-type–specific CpG sites.

### 2.7 Statistical analysis

Data are expressed as mean ± standard deviation (SD) for parametric data or as median and interquartile range (Q1-Q3) for nonparametric data. Normality was assessed using the Shapiro-Wilk test. The Wilcoxon matched-pairs test was used to compare paired groups, while the Kruskal-Wallis test with Dunn’s *post hoc* test was employed to compare multiple groups. Statistical analyses were conducted using GraphPad Prism 8 (GraphPad Software Inc., San Diego, CA, USA).

To investigate the potential clinical relevance of DNA methylation changes, we performed correlation analyses between the mean of β-values of intensities of significant DMRs and the corresponding clinical parameters measured. Specifically, we analyzed specific IgE and IgG antibody levels at baseline and/or follow-up. Correlations were calculated using Pearson’s correlation coefficient, and analyses were performed separately for methylation levels in both promoter-associated and gene regions. This approach allowed us to explore potential associations between methylation status and immunoglobulin profiles, providing insight into the relationship between epigenetic variation and allergic immune and tolerant responses.

## 3 Results

### 3.1 Study subjects and clinical data

CMAIE, CMANIE groups and control subjects exhibited similar demographic characteristics. Control individuals had negative serum test results, and throughout the study period, it was confirmed that they did not develop any allergic conditions. The only difference found between the groups was IgE serum levels, being statistically significantly different between the CMAIE and CMANIE groups as expected (**Table 1**).

**Table 1:** Demographic and clinical characteristics of individuals recruited to the study.

Variables	Control (*n* = 3)		CMAIE (*n* = 10)		CMANIE (*n* = 6)		*p*-value
Male, n (%)	2 (66)		3 (33)		3 (50)		N.S.
Age (months)^a^	4.0 (2-7)		7.5 (4.25-8.25)		5.0 (3.75-6)		N.S.
Exclusive breastfeeding (months)^b^	2.67 ± 4.62		5.70 ± 2.54		1.92 ± 1.11		N.S.
Atopic diseases, n (%)	0 (0)		4 (40)		1 (16)		N.S.
Symptoms							
Gastrointestinal, n (%)	0 (0)		2 (20)		6 (100)		*(Control *vs.* CMANIE) **(CMAIE *vs.* CMANIE)
Respiratory, n (%)	0 (0)		2 (20)		0 (0)		N.S.
Dermatological, n (%)	0 (0)		10 (100)		0 (0)		**(Control *vs.* CMANIE) ***(CMAIE *vs*. CMANIE)
Egg sensitization, n (%)	0 (0)		4 (40)		0 (0)		N.S.
Positive OFC, n (%)	NA		6 (60)		3 (50)		N.S.
sIgE (kU/L)^a^	T0	T1	T0	T1	T0	T1	
Cow’s milk protein	0.01 (0-0.27)	NA	1.65 (0.67-13.2)	0.83 (0.14-9.16)	0.01 (0.01-0.04)	0.02 (0.01-0.07)	*(CMAIE T0 *vs.* CMANIE T0)
α-lactoalbumin	0.01 (0-0.13)	NA	0.71 (0.01-11.28)	0.18 (0.01-9.3)	0 (0-0)	0.01 (0.01-0.01)	**(CMAIE T0 *vs.* CMANIE T0)
β-lactoglobulin	0.01 (0.01-0.31)	NA	0.74 (0.18-3.04)	0.3 (0.05-0.86)	0 (0-0.01)	0.02 (0.01-0.02)	**(CMAIE T0 *vs.* CMANIE T0) *(CMAIE T1 *vs.* CMANIE T1)
Casein	0.01 (0.01-0.02)	NA	0.24 (0.09-5.42)	0.23 (0.04-4.54)	0.005 (0-0.01)	0.01 (0.01-0.01)	*(CMAIE T0 *vs.* CMANIE T0)
sIgG Milk (kU/L)^a^	0.18 (0.01-4.88)	NA	0.23 (0.11-4.59)	0.15 (0.15-1.01)	0.15 (0.008-0.56)	0.15 (0.14-0.15)	N.S.

OFC, oral food challenge; n, number of cases; NA, not available; N.S., no significant differences.

^a^Median (25^th^-75^th^ Percentile); ^b^Mean ± SD; *p < 0.05; **p < 0.01; ***p < 0.001.

T0: baseline; T1: six months after the start of treatment.

Milk exposure tests (OFC) were conducted in both groups to assess tolerance. Tolerance was achieved in 4 participants (40%) in the CMAIE group and in 3 participants (50%) in the CMANIE group. In the CMAIE group, higher sensitization levels were closely associated with positive outcomes during the exposure tests. In all positive OFC cases, symptoms were mild, and the administration of adrenaline was not required.

A summary of the primary demographic and clinical characteristics of all three groups is provided in **Table 1**.

### 3.2 Quality control and DNA methylation analysis using EPIC arrays

All samples were evaluated by electrophoresis and classified as optimal for the assays. Additionally, all samples passed the quality control (corresponding to a Detected CpG % (0.05) ≥ 90%), making them suitable for subsequent methylation analysis by EPIC arrays.

Due to sample size limitation, we performed a power calculation based on effect size (Cohen’s D). Using a two-sample t-test with a significance level of 0.05 and assuming equal variances between groups, our study demonstrates adequate power (approximately 80% or higher) to detect large effect sizes (Cohen’s D ≥ 2) with the current sample sizes. This is further supported by the very low and thus highly significant adjusted p-values obtained in the comparisons.

### 3.3 CMA children present a different methylation profile according to pathological status

The initial differential methylation analysis revealed significant DMPs in the comparisons between CMAIE subjects at baseline and controls, with 5 hypermethylated and 3 hypomethylated DMPs identified.

Subsequently, differential methylation analysis was conducted at the region level (DMRs). As shown in **Table 2**, significant DMRs annotated to both genes and promoters were identified in all comparisons performed.

**Table 2:** Number of significant DMRs (adjusted p-value < 0.05) annotated to genes and promoters in the comparisons performed.

Comparison	DMRs annotated to promoters	DMRs annotated to genes
CMAIE T_1_ vs. CMAIE T_0_	33	25
CMAIE T_0_ vs. CMANIE T_0_	7	1
CMAIE T_0_ vs. Control	15	2
CMANIE T_0_ vs. Control	4	2
Tolerant T_0_ vs. Non-Tolerant T_0_	13	30
Tolerant T_1_ vs. Tolerant T_0_	57	71
Non-Tolerant T_1_ vs. Non-Tolerant T_0_	13	4

**Figure 2:**
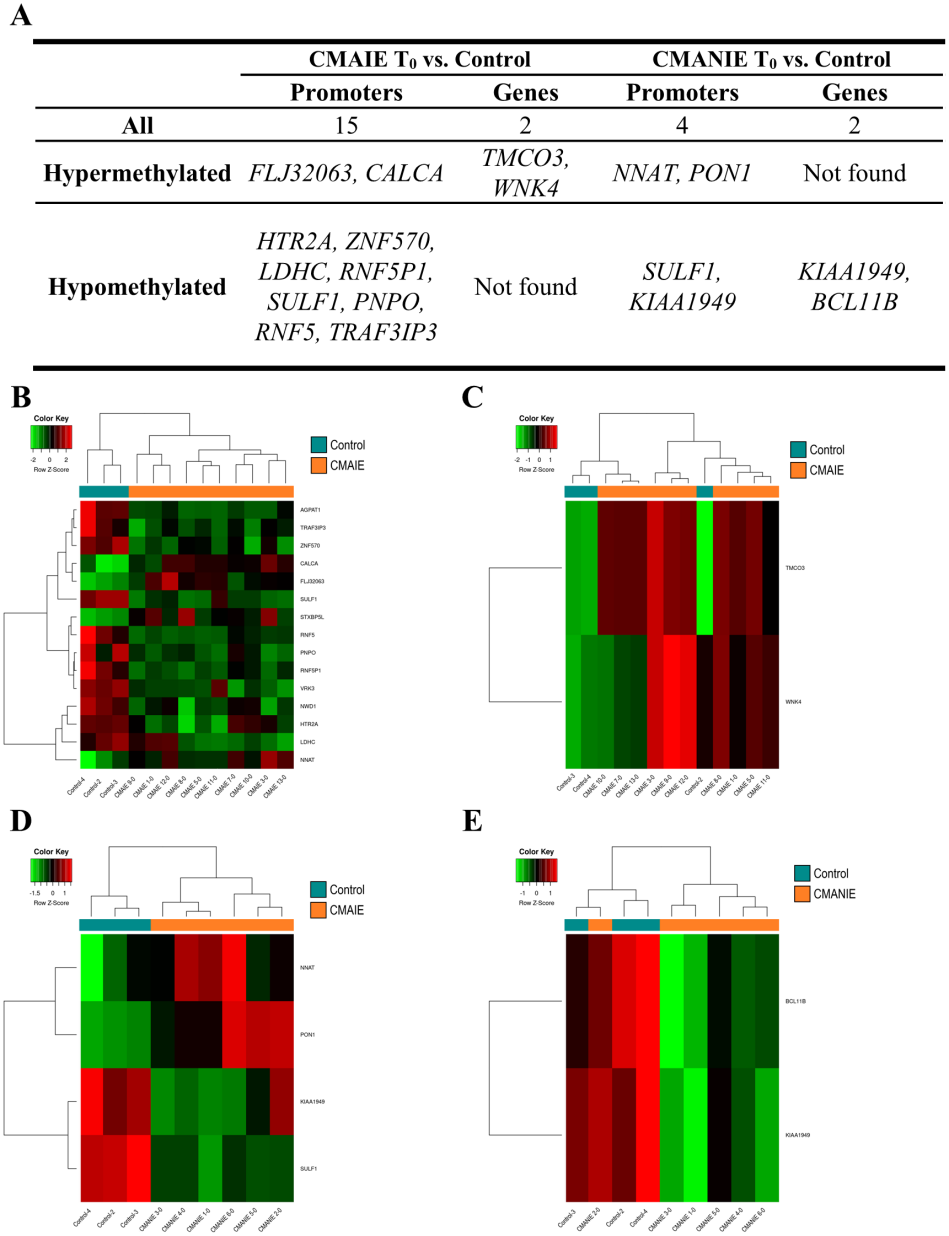
Methylation profile differentiates cow’s milk allergic patients from control individuals. **(A)** Top 10 DMRs identified in promoters and genes when comparing CMAIE and CMANIE groups at baseline to healthy controls. At baseline, both allergic groups (CMAIE and CMANIE) displayed distinct methylation profiles compared to control subjects. CMAIE comparison showed more differences in methylation profile than CMANIE. Heatmaps of significant DMRs found in promoters **(B)** and genes **(C)** comparing CMAIE patients at baseline to controls; and comparing DMRs observed in promoters **(D)** and genes **(E)** of CMANIE patients at T_0_ to controls. Heatmaps based on significant promoter- and gene-associated DMRs revealed clear sample clustering, with the strongest group separation observed in promoter-associated regions. Z-scores derived from beta methylation values are represented, where green indicates hypomethylation and red indicates hypermethylation. CMAIE, CMAIE patients at T_0_; CMANIE, CMANIE patients at T_0_.

Both groups of allergic children at baseline exhibited a distinct methylation profile compared to healthy controls (**Figure 2**). The CMAIE group demonstrated 2 hypermethylated DMRs in promoters and 13 hypomethylated DMRs, along with 2 hypermethylated DMRs associated with genes in comparison to controls (**Figure 2A**; **Supplementary Table S1**). Two heatmaps were generated using the mean methylation values of CpG probes located within the significant DMRs in promoters (**Figure 2B**) and genes (**Figure 2C**). In the heatmap generated from the DMRs detected in promoters, a perfect grouping of the samples into their respective groups was observed. In the heatmap generated from the DMRs detected in genes, two of the controls clustered correctly, separating from the remaining samples, but one control clustered with the CMAIE samples.

CMANIE group showed 2 hypermethylated and 2 hypomethylated DMR in promoters, and 2 hypomethylated DMRs associated to genes in comparison to controls (**Figure 2A**; **Supplementary Table S2**). The two heatmaps generated using the mean methylation values of CpG probes located in the significant DMRs detected in promoters (**Figure 2D**) and in genes (**Figure 2E**), showed that the samples clustered relatively well into their respective groups, with the heatmap generated from the DMRs obtained in promoters demonstrating a perfect grouping of the samples.

**Figure 3:**
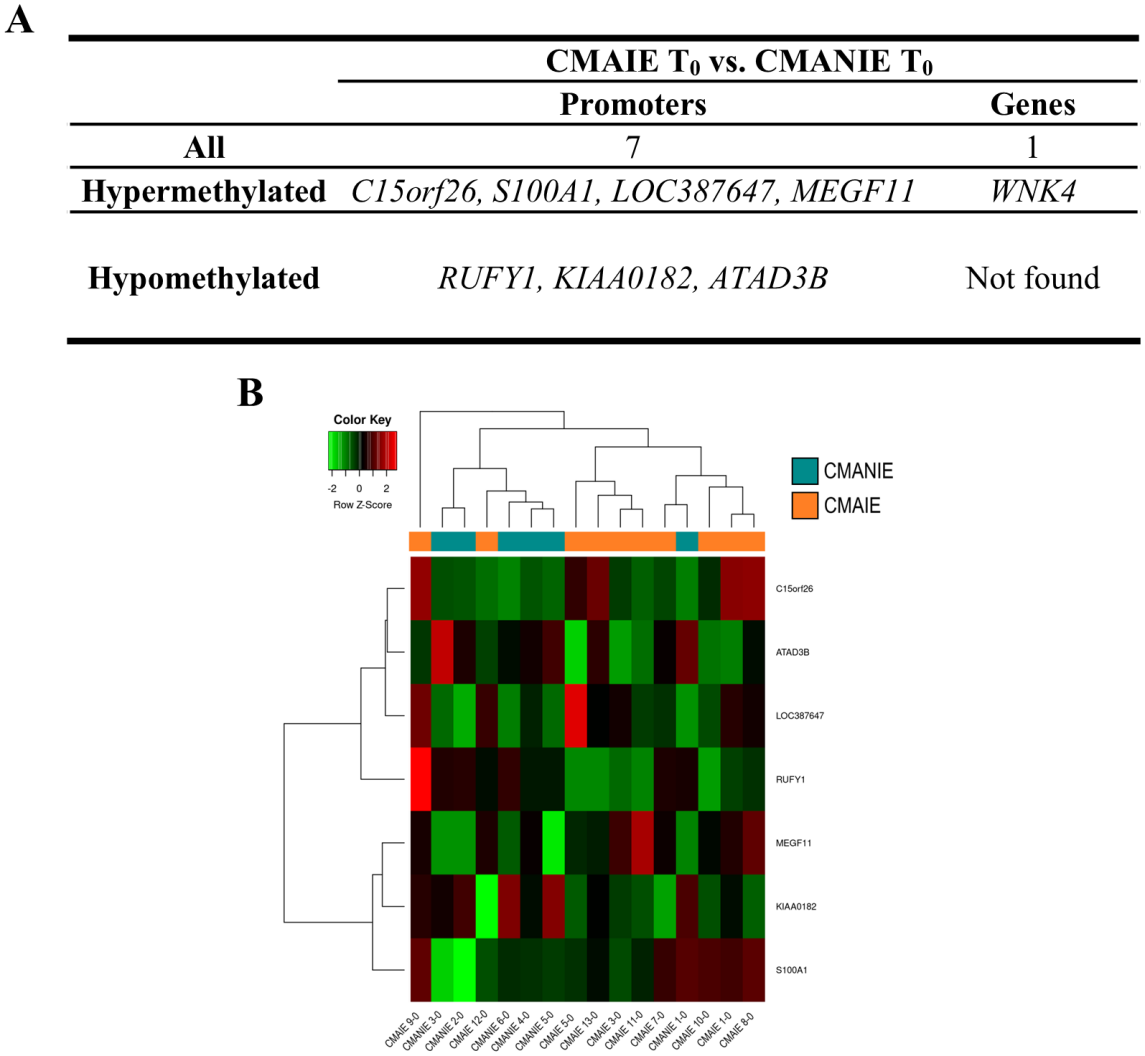
Distinct methylation profile in cow’s milk allergic patients at baseline. **(A)** A total of 7 significant DMRs were identified in promoter regions (4 hypermethylated and 3 hypomethylated in the CMAIE group), along with 1 additional hypermethylated region annotated to a gene. **(B)** Heatmap of promoter-associated DMRs shows partial separation of CMAIE and CMANIE groups. While several samples cluster according to their clinical classification, some overlap is observed, suggesting inter-individual heterogeneity in the baseline methylation profiles. Z-scores derived from beta methylation values are represented, where green indicates hypomethylation and red indicates hypermethylation. CMAIE, CMAIE patients at T_0_; CMANIE, CMANIE patients at T_0_.

On the other hand, we compared CMAIE and CMANIE groups at baseline, identifying a total of 7 significant DMRs annotated to promoters (4 hypermethylated and 3 hypomethylated in CMAIE group) and 1 hypermethylated to genes have been identified (**Figure 3A**; **Supplementary Table S3**). The heatmap performed with significant DMRs in promoters shows a relatively good separation of the samples into their respective groups, although several samples do not cluster correctly (**Figure 3B**).

### 3.4 Methylation changes in DNA could be associated to cow’s milk protein tolerance

Significant DMRs were also identified between T_0_ and T_1_ in the CMAIE group (**Table 2**). However, greater emphasis should be placed on interpreting these differences in relation to the oral food challenge results.

In the CMAIE group, 40% of patients (4 individuals) achieved tolerance following the exclusive diet intervention, as confirmed by negative results in the OFC. To further investigate differences in methylation profiles between tolerant and non-tolerant patients, as well as potential changes induced by the intervention, additional comparisons were solely conducted within the CMAIE cohort.

**Figure 4:**
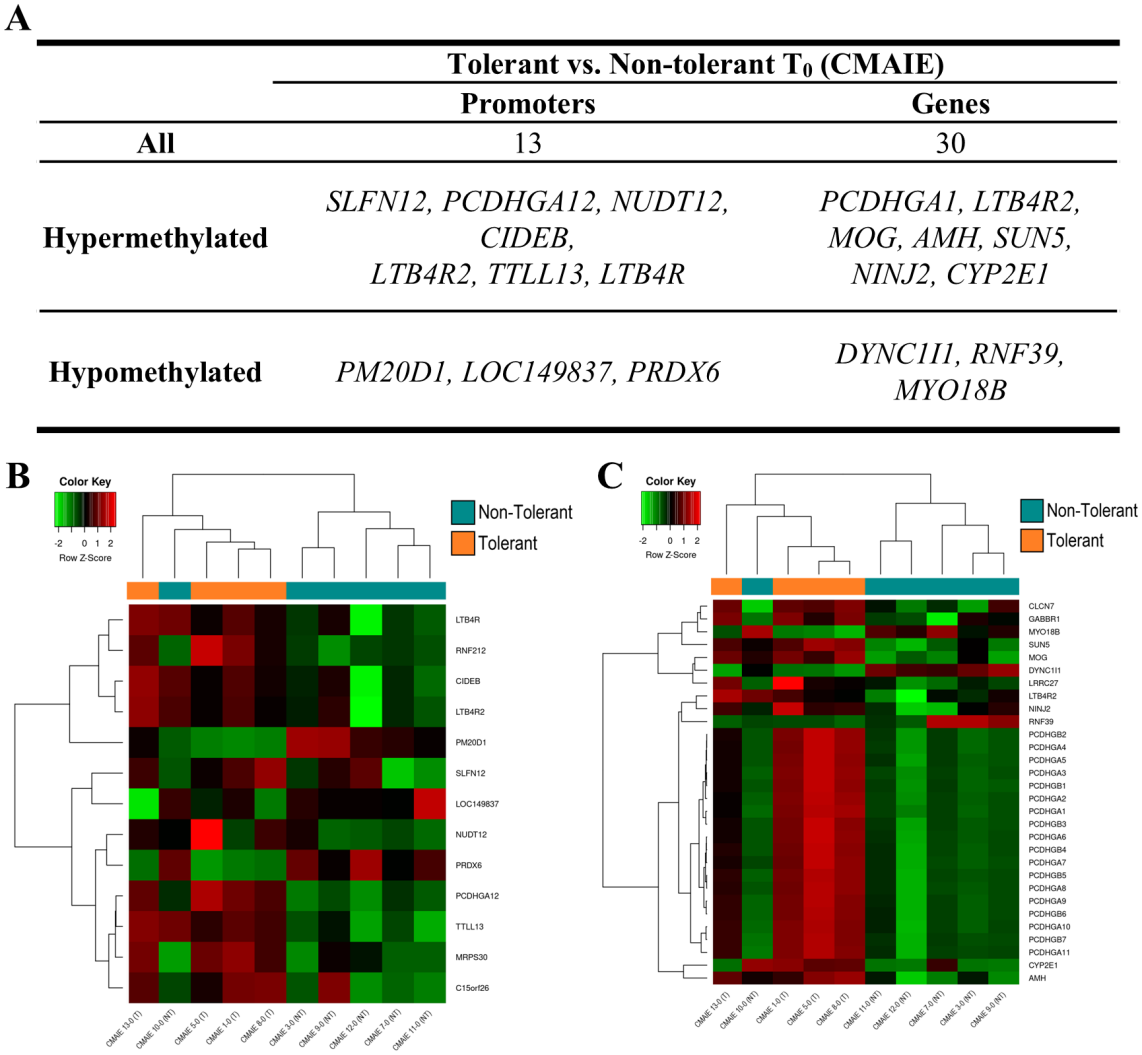
Differential DNA methylation distinguishes tolerant and non-tolerant CMAIE infants at baseline. **(A)** Top 10 most significant DMRs identified through mCSEA analysis, annotated to promoter regions and gene. More gene-associated DMRs were observed than promoter-associated DMRs. **(B, C)** Heatmaps based on average methylation values of CpG probes within significant promoter-associated **(B)** and gene-associated **(C)** DMRs. The methylation profiles demonstrate clear separation between tolerant and non-tolerant CMAIE infants, with only one non-tolerant subject (CMAIE 10-0) clustering with the tolerant group. Z-scores derived from beta methylation values are represented, where green indicates hypomethylation and red indicates hypermethylation. CMAIE: CMAIE patients.

On the one hand, we compared the tolerant and non-tolerant CMAIE patients at baseline, and in the mCSEA analysis we identified 13 significant DMRs annotated to promoters and 30 associated with genes (**Figure 4**, **Table 2** and **Supplementary Table S4**). **Figure 4A** shows the top 10 most significant DMRs identified in promoters and genes. Remarkably, there are several methylation marks in genes associated with leukotrienes and inflammation (LTB4R, LTBAR2). Additionally, two heatmaps were generated using the average methylation values of CpG probes located in the significant DMRs in promoters (**Figure 4B**) and genes (**Figure 4C**). The samples are relatively well-separated into their respective groups, with only one non-tolerant sample clustering with the tolerant ones in both heatmaps (CMAIE 10-0).

**Figure 5:**
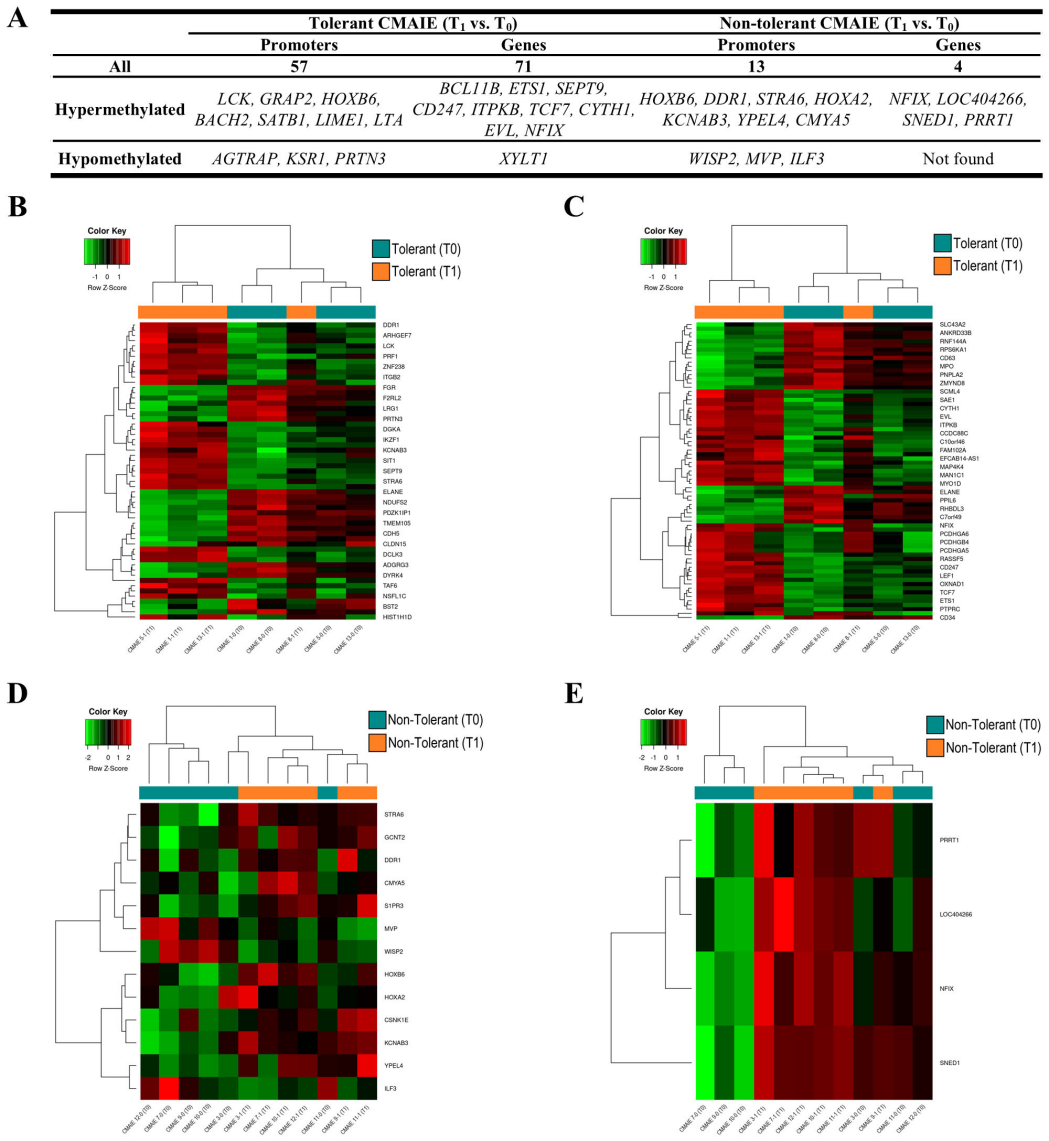
Tolerant children exhibit greater methylation profile changes compared to non-tolerant children after treatment. **(A)** Top 10 most significant DMRs identified in promoters and genes during the longitudinal comparison of tolerant and non-tolerant CMAIE patients before (T_0_) and after (T1) treatment. A total of 57 promoter-associated and 71 gene-associated DMRs were detected in tolerant patients, while only 13 and 4 were found in the non-tolerant group. **(B, C)** Heatmaps based on methylation of DMRs in promoters **(B)** and genes from tolerant patients. The methylation profiles largely distinguish samples by time point, with the exception of sample CMAIE 8-1. **(D, E)** Heatmaps of significant DMRs found in promoters **(D)** and genes **(E)** in the non-tolerant subgroup show limited differential methylation across time points. Z-scores derived from beta methylation values are represented, where green indicates hypomethylation and red indicates hypermethylation. CMAIE: CMAIE patients.

On the other hand, we compared tolerant patients before and after treatment to identify novel methylation-based biomarkers that could predict treatment success and distinguish successful outcomes from an ineffective exclusion diet (**Figure 5**). In the comparison between tolerant patients at T_0_ and T_1_, numerous methylation modifications were identified in both genes and promoters (**Table 2**). Specifically, 57 significant DMRs were annotated to promoters, while 71 were associated with genes (**Figure 5A**; **Supplementary Table S5**). Consistent with previous comparisons, the heatmaps (**Figures 5B**, **5C**) demonstrate that the samples are relatively well-separated into their respective groups. However, one sample for T_1_ (CMAIE 8-1) clusters with the T_0_ group in both heatmaps. In the comparison between T_0_ and T_1_ within the non-tolerant CMAIE subgroup, only a few differential methylation marks were identified (**Figure 5A**; **Table 2**). Specifically, 13 significant DMRs were annotated to promoters, while 4 were associated with genes (**Supplementary Table S6**). The heatmaps (**Figures 5D**, **5E**) generally show that samples cluster according to their respective time points (T_0_ or T_1_). However, in certain cases, such as the samples CMAIE 3–0 and CMAIE 3-1 (**Figure 5D**), the samples cluster together with their paired T_0_-T_1_ counterparts.

We also explore the potential clinical relevance of these epigenetic changes. Therefore, we performed correlation analyses between the methylation levels of DMRs found in promoters and genes and serum immunoglobulin levels, including both sIgE and sIgG (**Table 3**; **Supplementary Figure S1**). In non-tolerant individuals from CMAIE group, the methylation intensities linked to promoter of *HOXA2* consistently showed significant strong positive correlations with sIgE levels to whole milk, casein, α-lactoalbumin, and β-lactoglobulin at both baseline and after treatment (**Table 3**; **Supplementary Figure S1A**). These correlations remained stable over time, which could suggest a persistent epigenetic association with allergic sensitization that was not improved by treatment.

**Table 3:** Correlation between DNA methylation levels and serum immunoglobulin levels in tolerant and non-tolerant CMAIE individuals.

Serum immunoglobulin	NON-TOLERANT (T_1_ vs T_0_)				TOLERANT (T_1_ vs T_0_)															
	*HOXA2*				*ADGRG3*				*DYRK4*				*KSR1*				*TCF7*			
	T_0_		T_1_		T_0_		T_1_		T_0_		T_1_		T_0_		T_1_		T_0_		T_1_	
	r	*p*	r	*p*	r	*p*	r	*p*	r	*p*	r	*p*	r	*p*	r	*p*	r	*p*	r	*p*
Milk sIgE*	0.9877	0.0002	0.9948	< 0.0001	–	NS	–	NS	–	NS	–	NS	–	NS	0.9699	0.0301	–	NS	-0.9805	0.0195
Casein sIgE*	0.9213	0.009	0.9922	< 0.0001	–	NS	–	NS	–	NS	–	NS		NS	–	NS	–	NS	–	NS
α-lactoalbumin sIgE*	0.9348	0.0062	0.9545	0.0031	–	NS	–	NS	–	NS	0.9870	0.0130	–	NS	–	NS	–	NS	–	NS
β-lactoglobulin sIgE*	**–**	NS	0.9585	0.0025	–	NS	0.9609	0.0391	–	NS	–	NS	–	NS	0.9830	0.017	–	NS	-0.9567	0.0433
Milk sIgG*	**–**	NS	–	NS	0.9939	0.0061	–	NS	0.9794	0.0206	–	NS	0.9959	0.0041	–	NS	–	NS	–	NS

*kU/L; r, Pearson r coefficient; *p*, p-value; NS, no significant differences.

Significant correlations are highlighted in green (positive) and red (negative).

In contrast, tolerant individuals from CMAIE group displayed a dynamic pattern of correlation (**Table 3**; **Supplementary Figure S1B**). At T_0_, significant strong positive correlations were observed between IgG levels and the methylation intensities of promoters of *ADGRG3*, *DYRK4*, and *KSR1*, but no correlation with sIgE were observed. Following treatment, these correlations shifted toward a positive stronger association with sIgE levels for the same genes, along with *TCF7*, which showed a negative correlation with milk and β-lactoglobulin sIgE levels. These shifts could suggest a reprogramming of the epigenetic landscape accompanying the development of immune tolerance.

### 3.5 Methylation changes are associated with peripheral blood cell subpopulations and signaling pathways linked to immune response

**Figure 6:**
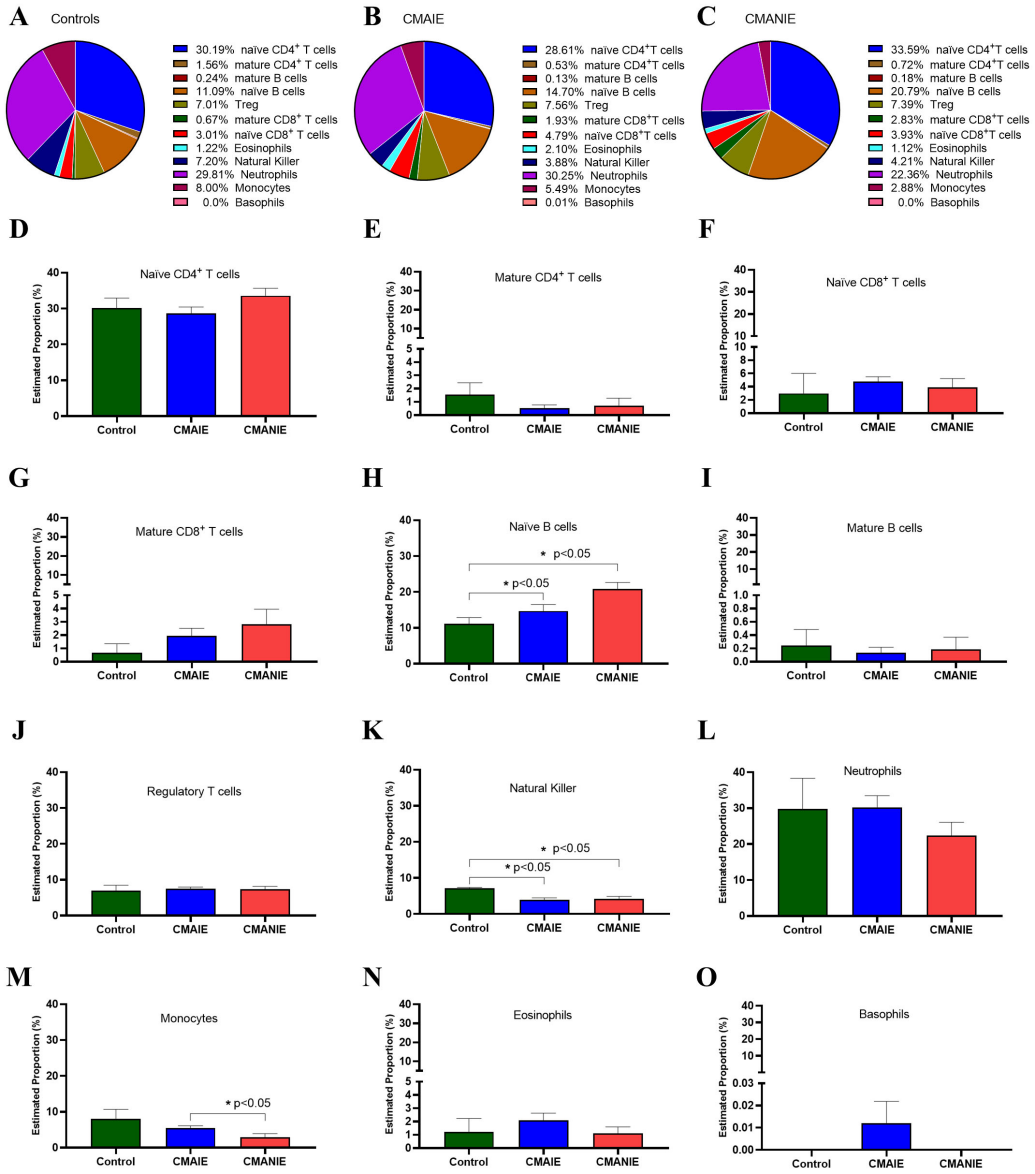
In silico estimation of immune cell composition linked to DMRs. Pie charts **(A-C)** and bar graphs **(D–O)** illustrate the average proportions of 12 immune cell types, including naïve CD4^+^ T cells **(D)**, mature CD4^+^ T cells **(E)**, naïve CD8^+^ T cells **(F)**, mature CD8^+^ T cells **(G)**, naïve B cells **(H)**, mature B cells **(I)**, regulatory T cells **(J)**, natural killer **(K)**, neutrophils **(L)**, monocytes **(M)**, eosinophils **(N)** and basophils **(O)** across the control, CMAIE, and CMANIE groups. No significant differences were observed in most immune cell populations, except for naïve B cells **(H)**, natural killer **(K)** and monocytes **(M)**. These findings suggest potential systemic immune involvement in CMA. Bars represent the mean ± SEM. Kruskal-Wallis test with Dunn’s *post hoc* test was employed to compare multiple groups,* indicate p<0.05.

Given that our DNA methylation analysis was performed using whole peripheral blood samples, we applied an *in silico* immune cell deconvolution approach to estimate the immune cell composition associated with the DMRs identified in this study. As shown in **Figure 6**, the majority of DMRs were linked to immune cell populations such as naïve CD4^+^ T cells as well as naïve B cells and neutrophils in all study groups (**Figures 6A–C**).

Specifically, we compared the distribution of each cell type among the three experimental groups (**Figures 6D–O**). Significant changes were observed in some of the analyzed populations, such as: i) the naïve B cell population, which differed significantly between the CMANIE group and controls (11.09 ± 3.04% vs. 20.79 ± 5.58%, *p* < 0.05, **Figure 6H**), as well as between CMAIE patients and control subjects (14.70 ± 8.02% vs. 20.79 ± 5.58%, *p* < 0.05, **Figure 6H**), showing higher proportions in both allergic groups compared to controls; ii) natural killer cells, whose percentages were significantly lower in both CMAIE (3.88 ± 2.66% vs. 7.20 ± 0.31%, *p* < 0.05, **Figure 6K**) and CMANIE (4.21 ± 1.99% vs. 7.20 ± 0.31%, *p* < 0.05, **Figure 6K**) groups compared to controls; and iii) monocytes, where the CMANIE group showed significantly lower percentages than CMAIE (2.88 ± 3.26% vs. 5.49 ± 2.77%, *p* < 0.05, **Figure 6M**). The remaining cell populations, including eosinophils and basophils, did not show significant differences among groups. These findings may suggest broader immune system involvement in CMA and are consistent with the systemic inflammatory and immunomodulatory profile characteristic of these allergic children.

Subsequently, a functional enrichment analysis was performed on the genes and promoters annotated to the significant DMRs, focusing on KEGG pathways and GO terms. **Table 4** presents the number of KEGG pathways and GO terms identified for each comparison. Significantly enriched GO terms and KEGG pathways were observed across all analyzed comparisons for both genes and promoters. However, in certain cases, the number of significant terms and pathways was very limited. Significance was defined as an adjusted p-value or FDR of less than 0.05.

**Table 4:** Number of Significantly Enriched KEGG Pathways and GO Terms Identified in the EnrichR Analysis Using Significant DMRs.

Comparison	Region	GO BP	GO MF	GO CC	KEGG
CMAIE T_0_*vs.* Control	Promoters	0	0	0	0
	Genes	4	7	3	0
CMANIE T_0_*vs.* Control	Promoters	57	5	2	0
	Genes	0	0	1	1
CMAIE T_0_*vs.* CMANIE T_0_	Promoters	10	3	0	0
	Genes	7	7	3	0
Tolerant T_0_*vs.* Non-tolerant T_0_	Promoters	48	6	0	0
	Genes	0	0	0	0
Tolerant T_1_*vs.* Tolerant T_0_	Promoters	9	7	13	11
	Genes	0	0	2	0
Non-tolerant T_1_*vs.* Non-tolerant T_0_	Promoters	0	0	0	0
	Genes	6	1	0	0

The terms included in this table are: GO Biological Process (GO BP), GO Molecular Function (GO MF), GO Cellular Component (GO CC), and KEGG pathways.

**Figure 7:**
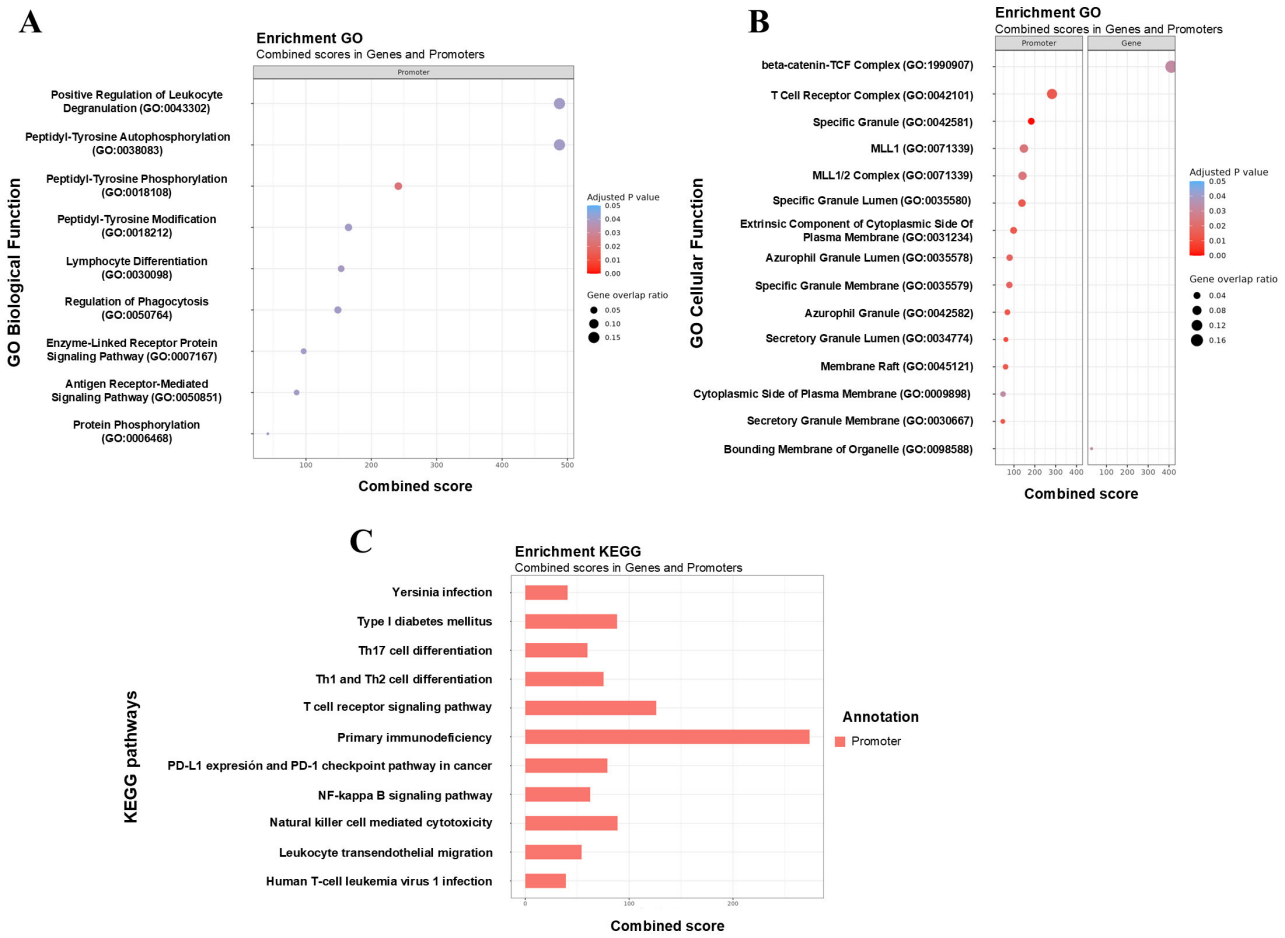
Functional enrichment of DMRs highlights key immune-related pathways associated with tolerance acquisition. **(A, B)** Gene Ontology (GO) enrichment analysis of differentially methylated promoters and gene identified in tolerant CMAIE patients before (T_0_) and after (T_1_) treatment. Significant enrichment was observed in biological processes linked to immune cell function, including positive regulation of leukocyte degranulation, lymphocyte differentiation, regulation of phagocytosis, and pathways related to the T cell receptor complex and secretory granule function. The combined score of enrichment, the adjusted p-value, and the gene overlap ratio are displayed. The gene overlap ratio is calculated by dividing the number of significant genes identified in the mCSEA analysis by the total number of genes associated with the respective GO term. **(C)** Barchart of KEGG terms combining scores of genes and promoters based on differential methylations found in tolerant CMAIE patients at baseline and after exclusion diet. KEGG pathway enrichment revealed additional immune and allergy-relevant pathways, such as Th1, Th2, and Th17 cell differentiation, T cell receptor signaling, and the NF-κB signaling pathway. .

It is important to emphasize the altered metabolic pathways in CMAIE-tolerant patients before and after treatment. By combining differentially methylated promoters and genes, several significantly altered GO pathways associated with immune cell functionality were identified. These included positive regulation of leukocyte degranulation, lymphocyte differentiation, regulation of phagocytosis, T cell receptor complex, and other pathways related to secretory granules (**Figures 7A, B**). Furthermore, KEGG enrichment analysis revealed additional allergic and immune-related pathways (**Figure 7C**), including Th17 cell differentiation, Th1 and Th2 cell differentiation, T cell receptor signaling pathway, and the NFκB signaling pathway.

However, the remaining pathways that were significantly altered between tolerant and non-tolerant individuals at different time points in the study primarily involved general signaling pathways with less direct relevance to the immune system and allergic response (data not shown).

## 4 Discussion

In this work, we provided evident differential methylation pattern in peripheral blood samples between individuals with IgE-mediated and non-IgE mediated CMA. These DNA methylation differences could have an important impact on several immune cells, being involved in the development of the disease or in the acquisition of tolerance. It has been previously demonstrated in animal models that changes in the methylation patterns of regulatory T cells (Treg) and dendritic cells could be associated with tolerance mechanisms (27, 28). Specifically, in this study, we assessed the distribution of immune cell populations *in silico* among CMAIE, CMANIE, and healthy control groups. Our findings revealed significant alterations in specific cell subsets (naïve B cells, natural killer cells and monocytes), suggesting broader immunological dysregulation associated with CMA. Naïve B cells were significantly increased in both allergic groups compared to controls. This expansion may reflect ongoing antigen exposure and immune activation, potentially contributing to the persistence of allergic sensitization in these children. Increased B cell activity, particularly in naïve and memory subtypes, has been associated with allergic diseases and IgE-mediated responses (29). Conversely, natural killer cells were significantly reduced in both allergic groups compared to controls, which may indicate an important role for these cells in modulating immune T2 responses. Reduced levels of invariant natural killer cells, particularly in peripheral blood, have been previously reported in individuals with CMA and may be associated with the development or severity of the allergy (30–32). Their reduction could contribute to an imbalanced immune environment that favors allergic inflammation. Regarding monocyte frequencies, significant differences were observed between the CMAIE and CMANIE groups. Monocytes are key regulators of both innate and adaptive immunity and can differentiate into macrophages or dendritic cells, thereby influencing T cell polarization (33). Notably, monocytes from infants with food allergies exhibit a hyperinflammatory phenotype, suggesting that the early onset of food allergy may be linked to dysregulated trained innate immune responses (34). This supports a potential role for monocytes in both IgE- and non-IgE-mediated responses in CMA. Collectively, our findings reinforce the concept that CMA is associated with systemic immunological alterations beyond classical effector pathways. The observed changes in naïve B cells, NK cells, and monocytes highlight a dysregulated immune profile that may influence both the severity and persistence of allergic disease.

In our study, differential methylation analysis at the position level revealed significant DMR differences. However, differential methylation analysis at the region level using mCSEA identified numerous significant DMRs associated with both genes and promoters across all comparisons. Our results show notable differences in DMRs linked to promoters and genes across all allergic profiles. In the case of patients with IgE-mediated CMA, while no direct associations with allergic diseases were found for most of the identified genes, previous studies for three genes were available. Notably, *LDHC* gene (lactate dehydrogenase C) has been reported as hypomethylated in CD4^+^ T cells of children with food allergy (35). Similarly, in the gene encoding the TRAF3IP3 protein (TNF receptor-associated factor 3 interacting protein 3), previous evidence has linked it to the regulation of the allergic immune response. Overexpression of this protein appears to play a significant role in the functionality of Treg cells and its involvement in the Th2 pathway of the allergic response (36). Another differentially methylated gene in the IgE allergic group is *TMCO3* (Transmembrane and Coiled-Coil Domains 3), which has also been associated with peanut allergies in a preliminary study (37).

On the other hand, in our study, we identified the differentially hypomethylated *BCL11B* in the CMANIE group. This gene is known for its role in T cell differentiation and type 2 innate lymphoid cells (ILC2) functionality. The differential methylation status of *BCL11B* could suggest a differential expression and a tendency toward the T2 inflammatory response, primarily through the increased production of cytokines such as IL-5 and IL-13 (38). In this study, the *BCL11B* gene is hypomethylated, suggesting overexpression of this gene. This finding aligns with studies that suggest the importance of the eosinophilic pathway in the development of non-IgE-mediated allergies (39); however, validation of *BCL11B* gene and protein expression levels is required to confirm these findings. The pathogenesis of non-IgE-mediated allergies is poorly understood, and no specific biomarkers have been identified to date. Previous studies have reported increased expression of inflammatory cytokines such as TNF-α, TGF-β1 receptor activity and TGF-β ligand expression in both tissue and peripheral blood monocytes from these patients. Additionally, elevated levels of inflammatory cytokines (IL-2, IL-8, IL-10, and IL-17) have also been observed in patients with symptoms consistent with food protein-induced enterocolitis syndrome (40, 41). Other studies suggest the possible involvement of the Th2 pathway in non-IgE-mediated allergies, with findings of elevated serum levels of cytokines such as IL-3, IL-5, and IL-13 in patients with this type of allergy (39). However, this is somewhat controversial, as other studies with the same objective have not found significant differences in the T2 response when compared to healthy individuals (42).

We also found some DMRs in promoters and genes between IgE and non-IgE-mediated CMA at baseline. Among the promoters that were differentially methylated, we must highlight *RUFY1* and *S100A1* in the CMAIE group, observing that *RUFY1* was hypomethylated, and previous studies have demonstrated changes in DNA methylation of this gene in children sensitized to airborne allergens (43). Additionally, *S100A1* was found to be hypermethylated in our sample, and similar studies have also identified hypermethylated regions in this gene in adolescents with multi-food allergies and in children with atopic dermatitis (44, 45).

Moreover, DMRs in promoters and genes have been identified in patients with IgE-mediated allergy after a cow’s milk restriction diet. Some of these genes have been previously linked to allergy, such as *ARHGEF7*, which was found to be differentially expressed in patients with lipid transfer protein (LTP) allergy (46). However, the significance of these findings becomes more pronounced when we consider tolerance acquisition. Previous studies have shown that methylation changes are associated with important genes involved in immune regulation and allergic responses, which may play a crucial role in the development of tolerance to allergens (8, 27, 28). In our study, we identified several genes and promoters that were differentially methylated between tolerant and non-tolerant individuals in the CMAIE group. Some of these genes have been previously linked to allergy and inflammation, and could suggest their potential involvement in the development of tolerance or persistence of allergic responses: *PM20D1* (44)*, SLFN12* (47)*, CIDEB* (48)*, LTB4R* and *LTB4R2* (49–51). Specifically, we found that *RNF9* was hypomethylated in tolerant individuals at baseline, similar to previous studies (44). This may be related to a differential expression, suggesting that *RNF39* could indicate an important role in the development of food allergy or immune tolerance. In the same line, *LTB4R* and *LTB4R2*, which are leukotriene B4 receptors (LTB4), have well-established roles in immunological diseases such as asthma. They are also important in the induction of the T2 response and eosinophil-mediated inflammation (50), therefore these receptors may play a significant role in the allergic mechanisms underlying food allergies, including potential mechanisms of tolerance or persistent allergic responses. Terawaki et al. concluded that *LTB4R*-deficient mice exhibited significantly attenuated hyperresponsiveness and eosinophilic inflammation compared to wild-type mice (52). Additionally, these deficient mice had reduced IgE levels in the plasma. In our study, *LTB4R* genes and promoters were hypermethylated in tolerant individuals at baseline, which appears to be consistent with the findings from these studies. It could be related to the involvement of *LTB4R* in the development of tolerance in food allergy patients. Similarly, *PCDHGA1* has been previously proposed as another potential biomarker for food allergy tolerance (51). This gene encodes a member of the protocadherin family, which is involved in cell-cell adhesion and signaling processes. Alterations in the expression or methylation of *PCDHGA1* could influence immune responses and contribute to the development or resolution of allergic reactions. In the context of food allergies, its differential methylation patterns could provide valuable insights into mechanisms of tolerance or allergic sensitization, further supporting its role as a potential biomarker.

Furthermore, we identified a methylation signature consisting of 57 DMRs annotated to promoters and 71 linked to genes in tolerant individuals, both before and after the restrictive diet. Notable genes associated with allergy, immune responses, and inflammation were found *(LCK, BACH2, SATB1, LIME1, KSR1, BCL11B, TCF1*, and *EVL)* (14, 53). Interestingly, some of these genes have been linked to tolerant responses related to Treg cells. For instance, *SATB1* is downregulated in FoxP3^+^ Tregs following subcutaneous and sublingual immunotherapy for grass pollen and is associated with clinical efficacy (54). Similarly, *TCF1* (encoded by the *TCF7* gene), which was hypermethylated in tolerant individuals after exclusion diet and negative correlated with milk and β-lactoglobulin sIgE levels, has been described as a critical determinant of Foxp3-dependent epigenetic changes (55), acting as a key positive regulator of chromatin accessibility in both conventional Foxp3^+^ cells. This could suggest that Foxp3 shapes the epigenetic identity of Treg cells primarily in an indirect manner, by modulating the activity of other key transcription factors such as *TCF1*. Moreover, *TCF7* is a key regulator in the Wnt signaling pathway, being essential for the development and maintenance of naïve and memory T cells and differentiation toward Th2 phenotype (56, 57). In our study, this gene was found to be hypermethylated in tolerant individuals after treatment, which may indicate a reduction in Th2 cell progression and differentiation, and consequently, a decrease in T2 response associated to CMA. Of particular interest is the *EVL* gene, which is regulated by IL-13 (58). Previous studies have shown that this gene is hypomethylated in patients with milk allergy compared to controls (15), likely due to high levels of IL-13. In our study, *EVL* was hypermethylated after diet in tolerant patients. Taken together, these findings suggest that the methylation of *EVL* gene may play a crucial role in the acquisition of tolerance in CMA and could serve as a potential biomarker of allergy remission. In allergic reactions, the innate immune system plays a key role in initiating and maintaining these responses. ILC2 release a series of cytokines that promote the inflammatory environment. It is known that the *BCL11B* regulates these ILC2, and a decrease in its expression or downregulation of *GATA3* and downstream genes (38), reduces inflammatory T2 responses. Tolerant individuals, after treatment, exhibited hypermethylation of *BCL11B*, which may indicate decreased expression and could partly explain the acquisition of tolerance, although this remains to be confirmed. However, in non-tolerant individuals, there was a persistent positive association between *HOXA2* methylation and sIgE levels indicates a possible role for this gene in maintaining an allergic phenotype. *HOXA2* is a homeobox-containing transcription factor primarily known for its role in embryonic patterning and craniofacial development (59). However, *HOXA2* has not been previously linked to allergic diseases or immune tolerance, but other members of the HOX gene family have demonstrated regulatory roles in type 2 immune responses (60). Based on the stable and positive correlations observed between *HOXA2* methylation and sIgE levels in non-tolerant individuals, *HOXA2* may play a role in sustaining a pro-allergic immune profile. However, further studies are needed to elucidate its potential involvement in allergic inflammation and its contribution to the establishment or persistence of immune tolerance.

Despite these encouraging findings, we acknowledge the limitations of our study, emphasizing the need for further validation of our results in a larger and independent cohort, particularly concerning gene and protein expression levels. Our study population consists of infants under 12 months of age, which poses challenges in itself for recruiting these patients and obtaining sufficient blood samples for analysis. For this reason, in our study, gene expression validation in patient samples was not performed, as it was not feasible to isolate specific immune cell subsets or to extract sufficient RNA for expression analysis. To address this limitation, we conducted a comprehensive search of the literature and public datasets to assess whether the DMRs observed in this study have previously been associated with gene expression changes in CMA or other food allergies. In this context, we found several publicly available epigenomic and transcriptomic datasets that support the biological relevance of our findings: i) in the public repository number GSE34639 (35, 61), which includes DNA methylation profiles of CD4^+^ T cells from 12-month-old infants with food allergy (including milk allergy), several of the key genes found in our study (*BCL11B*, *LTB4R*, *LTB4R2*, *TCF7*, and *EVL*) were also found to be differentially methylated between allergic and non-allergic groups; ii) in the public repositories numbers GSE114065 and GSE114134 (14), which contain matched methylation and RNA-seq data from naïve CD4^+^ T cells of infants with food allergy, overlapping signals that were observed in genes including *PCDHGA1*, *TCF7*, *LCK*, *BACH2*, and *EVL*, suggesting that epigenetic regulation of these loci may contribute to transcriptional changes during early-life allergic responses; and, lastly, iii) in the dataset numbers GSE189148 and GSE189149 (62), which profiled methylation and transcriptomic changes in CD4^+^ T cells from adolescents with peanut-only or multi-food allergy (including milk allergy), reported differential methylation and expression of *PM20D1*, *SLFN12*, *SATB1*, and *TCF7*, consistent with our findings in infants and reinforcing the hypothesis that these genes could be involved in the immunoregulatory mechanisms underpinning food allergy and tolerance. Together, this integrative evidence from independent studies and cohorts could support the biological and functional relevance of the epigenetic signals identified in our results, suggesting that methylation differences may influence gene expression patterns involved in the regulation of allergic inflammation and immune tolerance in the CMA context.

An important aspect of our study is that the inclusion criteria aligns with the diagnostic profiles of different allergy types, the diagnosis of non-IgE-mediated allergy may introduce some bias, as it relies on clinical diagnosis. To mitigate this, we recruited patients with clear clinical suspicion, excluding those with mild symptoms. Methylation changes serve as valuable long-term biomarkers; however, a direct, canonical relationship between methylation and gene expression is not always evident (63). Therefore, it is essential to explore these changes in relation to gene and protein expression and the underlying mechanisms involved. Nevertheless, these epigenetic changes hold significant promise as biomarkers for diagnosing IgE/non-IgE-mediated CMA and monitoring tolerance acquisition.

In conclusion, this study reports substantial changes in DNA methylation status between controls and children with CMA, and between IgE and non-IgE mediated CMA. These epigenetic changes were identified in key genes and promoters associated with allergy and inflammatory immune responses, particularly in the context of food allergies. Furthermore, in IgE-mediated cow’s milk allergy, we observed that genes such as *TCF7, EVL*, and *BCL11B* could be associated with allergy remission and tolerant responses, making them potential candidates as biomarkers for CMA and its prognosis regarding recovery.

## Data Availability

The datasets presented in this study can be found in online repositories. The names of the repository/repositories and accession number(s) can be found below: https://www.ncbi.nlm.nih.gov/geo/, https://www.ncbi.nlm.nih.gov/geo/query/acc.cgi?acc=GSE289021.
