# Supplementary material for: Differential DNA methylation in infants with IgE- and non-IgE-mediated cow’s milk allergy and its association with acquired tolerance
Source: Front Immunol. 2025 Nov 28;16:1571987. doi: 10.3389/fimmu.2025.1571987 (PMC12698524; doi:10.3389/fimmu.2025.1571987)
Supplement: Supplementary file 1 [file DataSheet1.docx]

Supplementary Material

| **PROMOTERS** | | | | | | **GENES** | | | | |  |
| --- | --- | --- | --- | --- | --- | --- | --- | --- | --- | --- | --- |
| **Name** | **ES** | **log2err** | **NES** | **pval** | **padj** | **Name** | **ES** | **log2err** | **NES** | **pval** | **padj** |
| *RNF5P1* | -0,71489 | 0,674963 | -2,38042 | 2,62E-07 | 0,000911 | *WNK4* | 0,742782 | 0,610527 | 2,257067 | 6,18E-06 | 0,041505 |
| *HTR2A* | -0,75928 | 0,704976 | -2,37773 | 1,07E-07 | 0,00081 | *TMCO3* | 0,582337 | 0,659444 | 2,264564 | 6,16E-07 | 0,008281 |
| *ZNF570* | -0,80998 | 0,704976 | -2,36128 | 1,15E-07 | 0,00081 |  |  |  |  |  |  |
| *SULF1* | -0,67037 | 0,690132 | -2,33465 | 2,27E-07 | 0,000911 |  |  |  |  |  |  |
| *RNF5* | -0,6894 | 0,643552 | -2,26285 | 1,62E-06 | 0,004029 |  |  |  |  |  |  |
| *LDHC* | -0,92649 | 0,690132 | -2,25636 | 1,4E-07 | 0,00081 |  |  |  |  |  |  |
| *PNPO* | -0,80584 | 0,643552 | -2,24362 | 1,29E-06 | 0,003735 |  |  |  |  |  |  |
| *TRAF3IP3* | -0,76952 | 0,627257 | -2,24331 | 2,28E-06 | 0,004954 |  |  |  |  |  |  |
| *VRK3* | -0,7182 | 0,593325 | -2,19905 | 1,21E-05 | 0,016211 |  |  |  |  |  |  |
| *NWD1* | -0,75737 | 0,57561 | -2,10867 | 1,58E-05 | 0,019636 |  |  |  |  |  |  |
| *AGPAT1* | -0,53043 | 0,57561 | -2,05301 | 2,71E-05 | 0,03148 |  |  |  |  |  |  |
| *FLJ32063* | 0,94978 | 0,610527 | 2,029532 | 4,18E-06 | 0,008087 |  |  |  |  |  |  |
| *STXBP5L* | 0,816905 | 0,610527 | 2,165395 | 7,4E-06 | 0,011708 |  |  |  |  |  |  |
| *NNAT* | 0,612459 | 0,593325 | 2,243034 | 1,05E-05 | 0,015277 |  |  |  |  |  |  |
| *CALCA* | 0,607449 | 0,610527 | 2,246609 | 5,03E-06 | 0,008756 |  |  |  |  |  |  |

# Supplementary Figures and Tables

**Table S1.** Significant DMRs identified in both promoters and gene in the CMAIE T_0_ vs. Control comparison.

ES, Enrichment Score; log2err, standard error; NES, Normalized Enrichment Score; pval, p-value; padj, Adjusted p-value.

**Table S2.** Significant DMRs identified in both promoters and gene in the CMANIE T_0_ vs. Control comparison.

| **PROMOTERS** | | | | | | **GENES** | | | | | |
| --- | --- | --- | --- | --- | --- | --- | --- | --- | --- | --- | --- |
| **Name** | **ES** | **log2err** | **NES** | **pval** | **padj** | **Name** | **ES** | **log2err** | **NES** | **pval** | **padj** |
| *SULF1* | -0,68638 | 0,674963 | -2,39515 | 3,87E-07 | 0,002244 | *KIAA1949* | -0,67727 | 0,74774 | -2,47288 | 1,26E-08 | 0,000169 |
| *KIAA1949* | -0,54805 | 0,610527 | -2,16381 | 5,64E-06 | 0,024536 | *BCL11B* | -0,48373 | 0,643552 | -2,06979 | 1,29E-06 | 0,008651 |
| *PON1* | 0,965065 | 0,674963 | 2,072056 | 2,77E-07 | 0,002244 |  |  |  |  |  |  |
| *NNAT* | 0,675528 | 0,719513 | 2,487067 | 3,16E-08 | 0,000549 |  |  |  |  |  |  |

ES, Enrichment Score; log2err, standard error; NES, Normalized Enrichment Score; pval, p-value; padj, Adjusted p-value.

**Table S3.** Significant DMRs identified in both promoters and gene in CMAIE and CMANIE at baseline comparison.

| **PROMOTERS** | | | | | | **GENES** | | | | | |
| --- | --- | --- | --- | --- | --- | --- | --- | --- | --- | --- | --- |
| **Name** | **ES** | **log2err** | **NES** | **pval** | **padj** | **Name** | **ES** | **log2err** | **NES** | **pval** | **padj** |
| *RUFY1* | -0,69867 | 0,593325 | -2,23315 | 9,68E-06 | 0,028806 | *WNK4* | 0,856369 | 0,801216 | 2,61288 | 6,44E-10 | 8,65E-06 |
| *KIAA0182* | -0,49767 | 0,593325 | -2,09541 | 9,61E-06 | 0,028806 |  |  |  |  |  |  |
| *ATAD3B* | -0,95518 | 0,593325 | -1,92456 | 9,94E-06 | 0,028806 |  |  |  |  |  |  |
| *LOC387647* | 0,952539 | 0,627257 | 2,039441 | 2,17E-06 | 0,012607 |  |  |  |  |  |  |
| *MEGF11* | 0,564641 | 0,593325 | 2,148814 | 1,44E-05 | 0,035857 |  |  |  |  |  |  |
| *C15orf26* | 0,944515 | 0,733762 | 2,23062 | 2,45E-08 | 0,000427 |  |  |  |  |  |  |
| *S100A1* | 0,744633 | 0,704976 | 2,42292 | 8,05E-08 | 0,0007 |  |  |  |  |  |  |

ES, Enrichment Score; log2err, standard error; NES, Normalized Enrichment Score; pval, p-value; padj, Adjusted p-value.

**Table S4.** Significant DMRs identified in both promoters and gene in the Tolerant vs. Non-Tolerant at baseline comparison.

| **PROMOTERS** | | | | | | **GENES** | | | | | |
| --- | --- | --- | --- | --- | --- | --- | --- | --- | --- | --- | --- |
| **Name** | **ES** | **log2err** | **NES** | **pval** | **padj** | **Name** | **ES** | **log2err** | **NES** | **pval** | **padj** |
| *PM20D1* | -0,99533 | 1,017545 | -2,3568 | 1,09E-15 | 1,9E-11 | *DYNC1I1* | -0,65503 | 0,627257 | -2,20698 | 3,39E-06 | 0,002396 |
| *PRDX6* | -0,87228 | 0,659444 | -2,27891 | 6,7E-07 | 0,00233 | *RNF39* | -0,60078 | 0,610527 | -2,17207 | 3,97E-06 | 0,002665 |
| *LOC149837* | -0,98074 | 0,74774 | -2,12842 | 1,3E-08 | 7,53E-05 | *MYO18B* | -0,54729 | 0,57561 | -2,10162 | 2,19E-05 | 0,012791 |
| *MRPS30* | 0,824692 | 0,593325 | 2,108075 | 1,42E-05 | 0,020004 | *GABBR1* | 0,389002 | 0,538434 | 1,795306 | 9,53E-05 | 0,043926 |
| *C15orf26* | 0,899197 | 0,593325 | 2,121708 | 7,7E-06 | 0,01217 | *SUN5* | 0,946778 | 0,57561 | 1,925136 | 2,96E-05 | 0,015928 |
| *RNF212* | 0,822531 | 0,593325 | 2,17648 | 1,5E-05 | 0,020004 | *NINJ2* | 0,471873 | 0,557332 | 1,947513 | 5,35E-05 | 0,027631 |
| *TTLL13* | 0,859868 | 0,610527 | 2,185757 | 4,25E-06 | 0,008222 | *CLCN7* | 0,491339 | 0,538434 | 1,954634 | 9,81E-05 | 0,043926 |
| *NUDT12* | 0,863551 | 0,659444 | 2,207406 | 9,2E-07 | 0,002666 | *LRRC27* | 0,513779 | 0,538434 | 1,960599 | 9,5E-05 | 0,043926 |
| *LTB4R* | 0,661456 | 0,610527 | 2,250829 | 4,75E-06 | 0,00827 | *AMH* | 0,868942 | 0,57561 | 2,074159 | 2,79E-05 | 0,015618 |
| *LTB4R2* | 0,733168 | 0,627257 | 2,324244 | 2,69E-06 | 0,005859 | *PCDHGA11* | 0,479343 | 0,659444 | 2,099791 | 8,74E-07 | 0,000652 |
| *CIDEB* | 0,707159 | 0,643552 | 2,357715 | 1,24E-06 | 0,003089 | *CYP2E1* | 0,778176 | 0,557332 | 2,109268 | 5,7E-05 | 0,028354 |
| *PCDHGA12* | 0,790521 | 0,690132 | 2,370379 | 2,2E-07 | 0,000957 | *MOG* | 0,630537 | 0,593325 | 2,166185 | 9,9E-06 | 0,006043 |
| *SLFN12* | 0,872065 | 0,788187 | 2,516255 | 1,79E-09 | 1,55E-05 | *LTB4R2* | 0,81522 | 0,610527 | 2,209679 | 6,04E-06 | 0,003865 |
|  |  |  |  |  |  | *PCDHGB7* | 0,495565 | 0,74774 | 2,227509 | 8,97E-09 | 7,09E-06 |
|  |  |  |  |  |  | *PCDHGA8* | 0,484685 | 0,801216 | 2,315238 | 5,76E-10 | 5,16E-07 |
|  |  |  |  |  |  | *PCDHGA10* | 0,507453 | 0,788187 | 2,320524 | 1,62E-09 | 1,36E-06 |
|  |  |  |  |  |  | *PCDHGB5* | 0,496695 | 0,814036 | 2,348027 | 3,54E-10 | 3,43E-07 |
|  |  |  |  |  |  | *PCDHGB6* | 0,504987 | 0,814036 | 2,349603 | 3,57E-10 | 3,43E-07 |
|  |  |  |  |  |  | *PCDHGA9* | 0,501073 | 0,839089 | 2,358404 | 1,04E-10 | 1,17E-07 |
|  |  |  |  |  |  | *PCDHGA7* | 0,491188 | 0,863415 | 2,360566 | 2,41E-11 | 3,23E-08 |
|  |  |  |  |  |  | *PCDHGB4* | 0,495244 | 0,851339 | 2,368157 | 3,03E-11 | 3,7E-08 |
|  |  |  |  |  |  | *PCDHGB3* | 0,497546 | 0,954542 | 2,449682 | 5,92E-14 | 9,95E-11 |
|  |  |  |  |  |  | *PCDHGA6* | 0,502931 | 0,943632 | 2,45504 | 1,42E-13 | 2,12E-10 |
|  |  |  |  |  |  | *PCDHGA5* | 0,502501 | 1,007318 | 2,481895 | 2,12E-15 | 4,08E-12 |
|  |  |  |  |  |  | *PCDHGB1* | 0,506093 | 1,105337 | 2,547574 | 2,18E-18 | 7,34E-15 |
|  |  |  |  |  |  | *PCDHGA4* | 0,507809 | 1,095929 | 2,547724 | 4,86E-18 | 1,31E-14 |
|  |  |  |  |  |  | *PCDHGB2* | 0,509315 | 1,047626 | 2,550087 | 1,43E-16 | 3,19E-13 |
|  |  |  |  |  |  | *PCDHGA3* | 0,517106 | 1,203975 | 2,625259 | 1,14E-21 | 5,11E-18 |
|  |  |  |  |  |  | *PCDHGA2* | 0,521684 | 1,237897 | 2,667017 | 5,65E-23 | 3,8E-19 |
|  |  |  |  |  |  | *PCDHGA1* | 0,519002 | 1,26274 | 2,691704 | 8,78E-24 | 1,18E-19 |

ES, Enrichment Score; log2err, standard error; NES, Normalized Enrichment Score; pval, p-value; padj, Adjusted p-value.

**Table S5.** Significant DMRs identified in promoters and gene from the comparison of tolerant CMAIE patients at T_1_ vs baseline (T_0_).

| **PROMOTERS** | | | | | | **GENES** | | | | | |
| --- | --- | --- | --- | --- | --- | --- | --- | --- | --- | --- | --- |
| **Name** | **ES** | **log2err** | **NES** | **pval** | **padj** | **Name** | **ES** | **log2err** | **NES** | **pval** | **padj** |
| *AGTRAP* | -0,90988 | 0,674963 | -2,25904 | 2,57E-07 | 0,000893 | *C7orf49* | -0,78888 | 0,610527 | -2,26482 | 4,7E-06 | 0,003947 |
| *IRF5* | -0,73937 | 0,593325 | -2,25119 | 8,97E-06 | 0,010223 | *RPS6KA1* | -0,62943 | 0,674963 | -2,24702 | 3,7E-07 | 0,000452 |
| *CLDN15* | -0,7986 | 0,593325 | -2,19974 | 8,21E-06 | 0,010223 | *PIWIL4* | -0,79505 | 0,557332 | -2,16539 | 3,23E-05 | 0,013996 |
| *FGR* | -0,68339 | 0,593325 | -2,18401 | 1,1E-05 | 0,011276 | *MPO* | -0,901 | 0,643552 | -2,16053 | 1,48E-06 | 0,001424 |
| *KSR1* | -0,55298 | 0,627257 | -2,17216 | 2,7E-06 | 0,004276 | *PNPLA2* | -0,72722 | 0,557332 | -2,12951 | 3,88E-05 | 0,015335 |
| *PRTN3* | -0,89761 | 0,627257 | -2,15993 | 2,6E-06 | 0,004276 | *CD34* | -0,73899 | 0,557332 | -2,1216 | 5,46E-05 | 0,018353 |
| *CDH5* | -0,74515 | 0,557332 | -2,14645 | 4,4E-05 | 0,026379 | *XYLT1* | -0,48595 | 0,690132 | -2,10674 | 1,4E-07 | 0,00021 |
| *BST2* | -0,84171 | 0,593325 | -2,14594 | 1,26E-05 | 0,011512 | *CLCF1* | -0,87537 | 0,593325 | -2,09908 | 8,24E-06 | 0,005749 |
| *CPNE6* | -0,80107 | 0,57561 | -2,14226 | 3,02E-05 | 0,020978 | *C17orf99* | -0,83924 | 0,557332 | -2,0919 | 3,7E-05 | 0,015335 |
| *MVB12A* | -0,75837 | 0,557332 | -2,11903 | 5,34E-05 | 0,029613 | *RAB31* | -0,54364 | 0,593325 | -2,06574 | 1,09E-05 | 0,006633 |
| *CTSZ* | -0,6698 | 0,538434 | -2,08652 | 8,02E-05 | 0,034017 | *CD63* | -0,79819 | 0,538434 | -2,05666 | 8,8E-05 | 0,028159 |
| *AZU1* | -0,86639 | 0,57561 | -2,08481 | 2,17E-05 | 0,017174 | *ANKRD33B* | -0,58235 | 0,557332 | -2,04623 | 4,6E-05 | 0,016952 |
| *NACC2* | -0,60621 | 0,557332 | -2,07592 | 5,62E-05 | 0,029613 | *PPIL6* | -0,6985 | 0,518848 | -2,04541 | 0,000181 | 0,040504 |
| *TMEM105* | -0,64562 | 0,538434 | -2,0633 | 6,89E-05 | 0,03246 | *IFFO1* | -0,62731 | 0,538434 | -2,03283 | 9,25E-05 | 0,028531 |
| *LINC01272* | -0,72293 | 0,518848 | -2,05381 | 0,00013 | 0,04435 | *ELANE* | -0,78551 | 0,518848 | -2,02399 | 0,000157 | 0,038264 |
| *KANSL1* | -0,72968 | 0,518848 | -2,03888 | 0,000158 | 0,048964 | *ZMYND8* | -0,53275 | 0,57561 | -2,02126 | 1,86E-05 | 0,009623 |
| *NDUFS2* | -0,61474 | 0,538434 | -2,03244 | 0,000114 | 0,041589 | *B4GALT5* | -0,66911 | 0,518848 | -2,01844 | 0,000212 | 0,044948 |
| *IL27* | -0,86723 | 0,557332 | -2,02198 | 5,87E-05 | 0,030025 | *RHBDL3* | -0,66699 | 0,518848 | -2,01204 | 0,000223 | 0,045534 |
| *DYRK4* | -0,83722 | 0,538434 | -2,01461 | 0,00012 | 0,041589 | *PPCDC* | -0,65377 | 0,538434 | -2,00143 | 0,000119 | 0,031954 |
| *ELANE* | -0,85069 | 0,518848 | -1,98343 | 0,000143 | 0,046981 | *MXD3* | -0,90045 | 0,538434 | -2,0009 | 6,65E-05 | 0,021781 |
| *ADGRG3* | -0,92677 | 0,557332 | -1,97696 | 3,39E-05 | 0,021822 | *RNF144A* | -0,64729 | 0,518848 | -1,98159 | 0,000168 | 0,038874 |
| *PDZK1IP1* | -0,91144 | 0,538434 | -1,94425 | 7,95E-05 | 0,034017 | *PFDN5* | -0,85847 | 0,538434 | -1,9799 | 0,000107 | 0,030202 |
| *F2RL2* | -0,90448 | 0,538434 | -1,92941 | 0,000114 | 0,041589 | *CSTF3-AS1* | -0,88888 | 0,518848 | -1,97518 | 0,000134 | 0,034517 |
| *LRG1* | -0,93059 | 0,538434 | -1,84327 | 0,000117 | 0,041589 | *SLC43A2* | -0,50679 | 0,518848 | -1,91691 | 0,000163 | 0,03879 |
| *SEPT9* | 0,385311 | 0,57561 | 1,839456 | 1,71E-05 | 0,014154 | *FNDC3B* | -0,44688 | 0,518848 | -1,86091 | 0,000206 | 0,044591 |
| *DCLK3* | 0,931965 | 0,518848 | 1,859039 | 0,000155 | 0,048964 | *JAZF1* | -0,42639 | 0,518848 | -1,79386 | 0,000173 | 0,039503 |
| *SIT1* | 0,953331 | 0,57561 | 1,90166 | 2,96E-05 | 0,020978 | *PCDHGA5* | 0,332954 | 0,498493 | 1,61233 | 0,000251 | 0,04854 |
| *TFAP2B* | 0,914528 | 0,538434 | 1,936005 | 6,32E-05 | 0,031418 | *CDH4* | 0,317235 | 0,57561 | 1,640594 | 2,83E-05 | 0,01266 |
| *ARHGEF7* | 0,500998 | 0,538434 | 1,955359 | 6,9E-05 | 0,03246 | *PCDHGA8* | 0,361396 | 0,518848 | 1,696796 | 0,000194 | 0,042745 |
| *TOX2* | 0,517447 | 0,518848 | 1,963359 | 0,000153 | 0,048964 | *PCDHGA7* | 0,364696 | 0,518848 | 1,721444 | 0,000165 | 0,03879 |
| *C10orf99* | 0,928984 | 0,57561 | 1,966608 | 2,68E-05 | 0,020274 | *PCDHGA6* | 0,359263 | 0,538434 | 1,721488 | 0,000108 | 0,030202 |
| *KCNAB3* | 0,777865 | 0,518848 | 1,970951 | 0,000162 | 0,049568 | *PCDHGB4* | 0,36759 | 0,538434 | 1,725154 | 0,000116 | 0,031705 |
| *ITGB2* | 0,567095 | 0,538434 | 1,977921 | 0,000119 | 0,041589 | *UBAC2* | 0,410783 | 0,518848 | 1,784848 | 0,000151 | 0,037642 |
| *NSFL1C* | 0,826025 | 0,518848 | 1,994013 | 0,000139 | 0,046405 | *CCDC88C* | 0,390248 | 0,557332 | 1,786578 | 4,67E-05 | 0,016952 |
| *CD3D* | 0,864044 | 0,538434 | 2,012107 | 7,45E-05 | 0,033229 | *MYO1D* | 0,429185 | 0,518848 | 1,827214 | 0,000227 | 0,045534 |
| *HIST1H1D* | 0,871608 | 0,557332 | 2,029721 | 5,57E-05 | 0,029613 | *MAP4K4* | 0,456288 | 0,538434 | 1,840842 | 9,34E-05 | 0,028531 |
| *ADORA2A* | 0,619146 | 0,538434 | 2,037045 | 9,86E-05 | 0,038095 | *PDE7A* | 0,564183 | 0,518848 | 1,906887 | 0,000214 | 0,044948 |
| *LY86* | 0,912744 | 0,593325 | 2,044307 | 1,36E-05 | 0,011834 | *C10orf46* | 0,903415 | 0,518848 | 1,920836 | 0,000148 | 0,037634 |
| *GALNT6* | 0,644459 | 0,538434 | 2,050886 | 8,22E-05 | 0,034032 | *PRKCH* | 0,442952 | 0,57561 | 1,925357 | 2,14E-05 | 0,009896 |
| *ZNF238* | 0,726026 | 0,538434 | 2,060514 | 8,93E-05 | 0,035293 | *SPINK2* | 0,905773 | 0,518848 | 1,925851 | 0,000122 | 0,032259 |
| *ALPK2* | 0,788214 | 0,538434 | 2,061491 | 7,19E-05 | 0,032911 | *UCP3* | 0,866724 | 0,498493 | 1,92768 | 0,000253 | 0,04854 |
| *ZAP70* | 0,638962 | 0,538434 | 2,066325 | 8,65E-05 | 0,034982 | *EFCAB14-AS1* | 0,870685 | 0,518848 | 1,936491 | 0,000227 | 0,045534 |
| *PRF1* | 0,81894 | 0,557332 | 2,075027 | 3,24E-05 | 0,021706 | *ARHGEF7* | 0,443807 | 0,593325 | 1,95297 | 1,2E-05 | 0,00672 |
| *TSPAN14* | 0,6575 | 0,557332 | 2,092385 | 4,15E-05 | 0,025759 | *SNED1* | 0,490419 | 0,557332 | 1,968236 | 3,79E-05 | 0,015335 |
| *DDR1* | 0,545309 | 0,593325 | 2,106568 | 1,17E-05 | 0,011325 | *SCML4* | 0,624488 | 0,498493 | 1,983101 | 0,000257 | 0,048643 |
| *DGKA* | 0,675298 | 0,557332 | 2,122752 | 5,56E-05 | 0,029613 | *IKZF1* | 0,597602 | 0,557332 | 2,019839 | 5,12E-05 | 0,018112 |
| *LZTFL1* | 0,619188 | 0,593325 | 2,159612 | 8,6E-06 | 0,010223 | *MGAT4A* | 0,664748 | 0,518848 | 2,021033 | 0,000232 | 0,045932 |
| *IKZF1* | 0,689585 | 0,593325 | 2,194491 | 9,4E-06 | 0,010223 | *VPS13D* | 0,479453 | 0,610527 | 2,029694 | 5,58E-06 | 0,004407 |
| *STRA6* | 0,675728 | 0,610527 | 2,210283 | 5,24E-06 | 0,007594 | *OXNAD1* | 0,95763 | 0,643552 | 2,036108 | 1,3E-06 | 0,001344 |
| *TAF6* | 0,699753 | 0,627257 | 2,262917 | 2,5E-06 | 0,004276 | *MAN1C1* | 0,481206 | 0,593325 | 2,04869 | 7,68E-06 | 0,005732 |
| *BACH2* | 0,546443 | 0,690132 | 2,263802 | 1,75E-07 | 0,000762 | *MME* | 0,787783 | 0,538434 | 2,051546 | 9,61E-05 | 0,028701 |
| *LTA* | 0,8031 | 0,643552 | 2,279255 | 1,08E-06 | 0,002344 | *FAM102A* | 0,619936 | 0,538434 | 2,052003 | 0,0001 | 0,029337 |
| *SATB1* | 0,800788 | 0,659444 | 2,297079 | 6,88E-07 | 0,001709 | *MGC12982* | 0,830044 | 0,557332 | 2,064853 | 5,41E-05 | 0,018353 |
| *LIME1* | 0,823208 | 0,659444 | 2,315796 | 6,19E-07 | 0,001709 | *SAE1* | 0,848607 | 0,57561 | 2,111033 | 1,94E-05 | 0,009639 |
| *HOXB6* | 0,731674 | 0,74774 | 2,520214 | 1,36E-08 | 7,86E-05 | *UBASH3A* | 0,731872 | 0,57561 | 2,13049 | 1,78E-05 | 0,009592 |
| *LCK* | 0,853973 | 0,875325 | 2,684405 | 1,19E-11 | 1,03E-07 | *RAD54B* | 0,77204 | 0,557332 | 2,130568 | 4,62E-05 | 0,016952 |
| *GRAP2* | 0,763285 | 0,875325 | 2,732853 | 9,47E-12 | 1,03E-07 | *PRRT1* | 0,673915 | 0,57561 | 2,140061 | 2,06E-05 | 0,009874 |
|  |  |  |  |  |  | *SLC7A6* | 0,743393 | 0,593325 | 2,164028 | 1,16E-05 | 0,00672 |
|  |  |  |  |  |  | *RASSF5* | 0,608294 | 0,593325 | 2,165324 | 8,91E-06 | 0,005749 |
|  |  |  |  |  |  | *NFIX* | 0,516106 | 0,674963 | 2,177048 | 2,61E-07 | 0,000351 |
|  |  |  |  |  |  | *SEPT9* | 0,446661 | 0,839089 | 2,189062 | 8,58E-11 | 3,84E-07 |
|  |  |  |  |  |  | *PTPRC* | 0,664069 | 0,593325 | 2,210815 | 8,99E-06 | 0,005749 |
|  |  |  |  |  |  | *SATB1* | 0,75787 | 0,627257 | 2,259627 | 2,69E-06 | 0,002413 |
|  |  |  |  |  |  | *LEF1* | 0,641606 | 0,643552 | 2,264829 | 9,76E-07 | 0,001093 |
|  |  |  |  |  |  | *CYTH1* | 0,56472 | 0,704976 | 2,278298 | 1,01E-07 | 0,000194 |
|  |  |  |  |  |  | *ITPKB* | 0,553486 | 0,761461 | 2,3431 | 6,71E-09 | 1,8E-05 |
|  |  |  |  |  |  | *EVL* | 0,650974 | 0,704976 | 2,366306 | 1,18E-07 | 0,000199 |
|  |  |  |  |  |  | *TCF7* | 0,723761 | 0,719513 | 2,409621 | 3,02E-08 | 6,77E-05 |
|  |  |  |  |  |  | *CD247* | 0,654579 | 0,826657 | 2,540495 | 1,37E-10 | 4,6E-07 |
|  |  |  |  |  |  | *ETS1* | 0,638133 | 0,863415 | 2,577277 | 2,56E-11 | 1,72E-07 |
|  |  |  |  |  |  | *BCL11B* | 0,690748 | 1,123915 | 2,95021 | 6,41E-19 | 8,61E-15 |

ES, Enrichment Score; log2err, standard error; NES, Normalized Enrichment Score; pval, p-value; padj, Adjusted p-value.

**Table S6.** Significant promoter and gene DMRs in non-tolerant CMAIE patients at T_1_ compared to baseline (T_0_).

| **PROMOTERS** | | | | | | **GENES** | | | | | |
| --- | --- | --- | --- | --- | --- | --- | --- | --- | --- | --- | --- |
| **Name** | **ES** | **log2err** | **NES** | **pval** | **padj** | **Name** | **ES** | **log2err** | **NES** | **pval** | **padj** |
| *MVP* | -0,74869 | 0,610527 | -2,28914 | 6,75E-06 | 0,019557 | *LOC404266* | 0,537345 | 0,610527 | 2,127693 | 6,41E-06 | 0,029928 |
| *WISP2* | -0,8482 | 0,643552 | -2,24017 | 1,62E-06 | 0,006615 | *SNED1* | 0,527256 | 0,610527 | 2,134858 | 6,68E-06 | 0,029928 |
| *ILF3* | -0,69926 | 0,57561 | -2,18786 | 1,6E-05 | 0,028233 | *PRRT1* | 0,695758 | 0,593325 | 2,221273 | 1,11E-05 | 0,03727 |
| *CMYA5* | 0,894405 | 0,57561 | 2,09548 | 1,79E-05 | 0,028233 | *NFIX* | 0,544246 | 0,719513 | 2,313756 | 3,32E-08 | 0,000445 |
| *KCNAB3* | 0,828738 | 0,593325 | 2,102923 | 1,1E-05 | 0,023888 |  |  |  |  |  |  |
| *CSNK1E* | 0,700122 | 0,557332 | 2,118131 | 3,39E-05 | 0,045394 |  |  |  |  |  |  |
| *S1PR3* | 0,694337 | 0,57561 | 2,182313 | 2,03E-05 | 0,029458 |  |  |  |  |  |  |
| *GCNT2* | 0,631381 | 0,57561 | 2,194116 | 1,73E-05 | 0,028233 |  |  |  |  |  |  |
| *YPEL4* | 0,797922 | 0,593325 | 2,194135 | 9,86E-06 | 0,023888 |  |  |  |  |  |  |
| *DDR1* | 0,573689 | 0,643552 | 2,228088 | 1,9E-06 | 0,006615 |  |  |  |  |  |  |
| *STRA6* | 0,692365 | 0,643552 | 2,283952 | 1,72E-06 | 0,006615 |  |  |  |  |  |  |
| *HOXA2* | 0,734086 | 0,643552 | 2,307247 | 1,72E-06 | 0,006615 |  |  |  |  |  |  |
| *HOXB6* | 0,698563 | 0,690132 | 2,42758 | 1,97E-07 | 0,003419 |  |  |  |  |  |  |

ES, Enrichment Score; log2err, standard error; NES, Normalized Enrichment Score; pval, p-value; padj, Adjusted p-value.


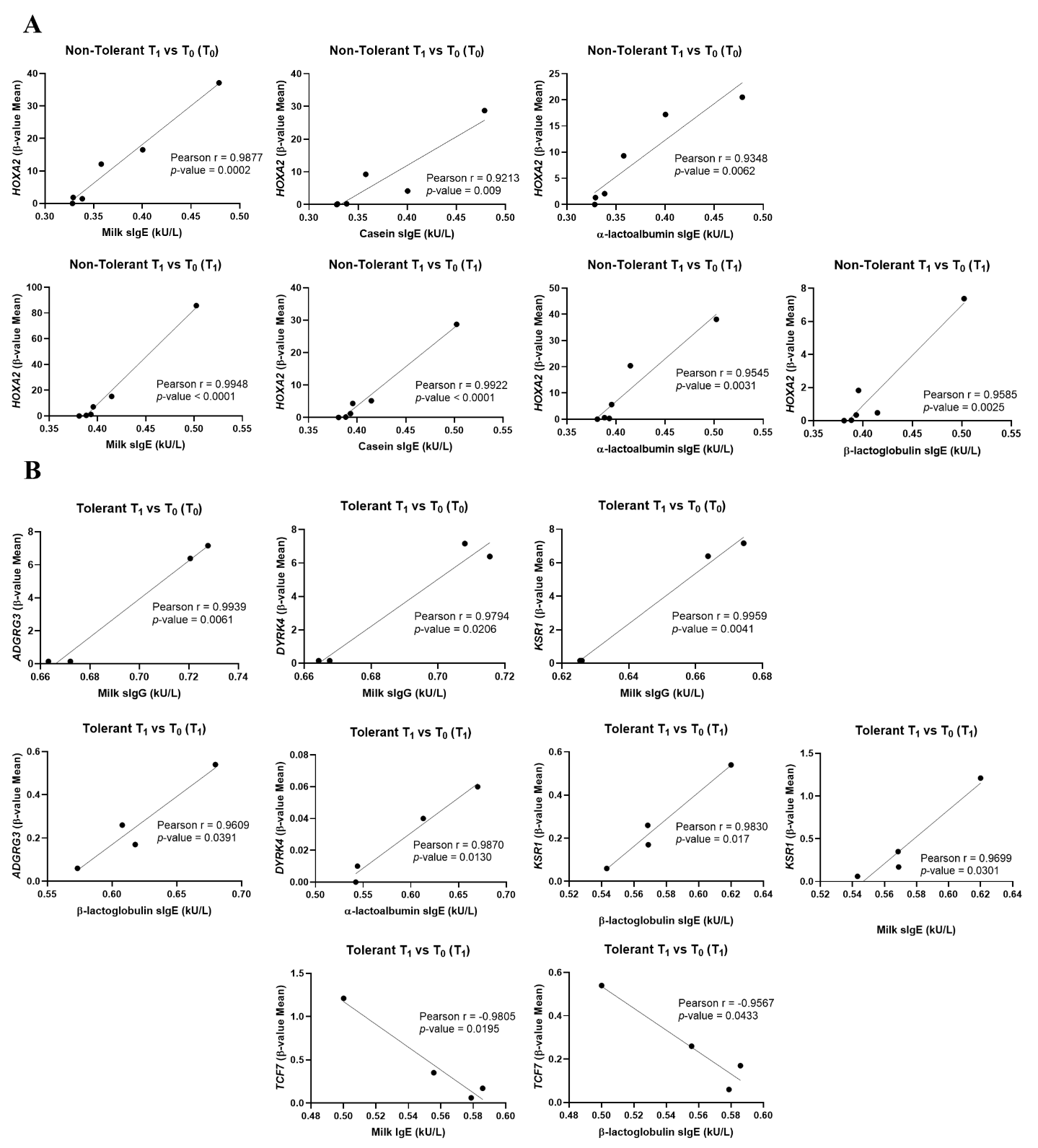


**Figure S1.** **Correlation between DNA methylation levels and serum immunoglobulin levels in CMAIE individuals.** **(A)** In non-tolerant individuals, strong and significant positive correlations were observed between methylation levels at the *HOXA2* promoter and serum sIgE levels to whole milk, casein, α-lactalbumin, and β-lactoglobulin at both baseline (T₀) and after treatment (T₁). **(B)** Tolerant individuals showed, at baseline (T₀), significant positive correlations between methylation levels of *ADGRG3*, *DYRK4*, and *KSR1* promoters and serum IgG levels. After treatment (T₁), the correlations shifted, showing stronger positive associations with sIgE levels for the same genes. Additionally, *TCF7* promoter methylation levels exhibited significant negative correlations with sIgE levels to milk and β-lactoglobulin at T₁. Pearson correlation coefficients (r) and p-values are indicated in each plot.
